# RILPL2 suppresses metabolic reprogramming and progression of cervical cancer by attenuating LDHA protein stability and inhibiting H3K18 lactylation

**DOI:** 10.1038/s41419-026-08808-9

**Published:** 2026-05-04

**Authors:** Yujing Shi, Zhaoyue Zhang, Jin Liu, Caiqiang Zhu, Gefenqiang Shen, Meng Tian, Liang Liang, Jinhui Liu, Xiaoke Di

**Affiliations:** 1 Department of Oncology, Jurong hospital affiliated to Jiangsu university, Zhenjiang, Jiangsu province China; 2 Department of Oncology, Jiangsu province (Suqian) Hospital, Suqian, China; 3 Department of Radiation Oncology, The First Affiliated Hospital of Nanjing Medical University, Nanjing, China; 4 Department of Gynecology, The First Affiliated Hospital of Nanjing Medical University, Nanjing, China

**Keywords:** Cancer metabolism, Ubiquitylation, Cell growth, Tumour-suppressor proteins

## Abstract

Cervical cancer (CC) is a prevalent malignancy among women worldwide with considerable incidence and mortality. Recent studies have suggested that the Rab-interacting lysosomal protein-like 2 (RILPL2) acts as a tumor suppressor and plays an inhibitory role in multiple human cancers. However, the potential effect of RILPL2 in CC remains unclear. In our investigation, we found that RILPL2 was downregulated in CC samples and was associated with a favorable outcome. Further findings indicated the interaction between RILPL2 and lactate dehydrogenase A (LDHA), a crucial player regulating glycolysis. Mechanistically, RILPL2 reduced LDHA stability by recruiting TRIM21 to facilitate K48-linked ubiquitination chains of LDHA and promoting LDHA degradation, thereby blocking glycolytic reprogramming and, in turn, inhibiting CC progression and development. Moreover, RILPL2-mediated inhibition of the glycolytic pathway could restrain lactate production, which abolished H3K18 lactylation to induce the downregulation of SOX9 and SMYD2. Consequently, our results suggested that RILPL2 may serve as a potential therapeutic target for the treatment of CC.

## Introduction

Cervical cancer (CC) represents a substantial global public health challenge, with a large number of new cases and deaths occurring globally each year [1–3]. The occurrence of CC is notably elevated in low- and middle-income nations, where the availability of screening and vaccination initiatives is constrained [4, 5]. Despite advancements in early detection and treatment, including surgery, radiotherapy, and chemotherapy, the outlook for patients diagnosed at this stage continues to be unfavorable, highlighting the critical necessity for the development of novel therapeutic strategies [6–8].

Accumulating research has underscored the essential effect of metabolic reprogramming in tumor progression, suggesting that altered cellular metabolism is not merely a consequence of tumorigenesis but may actively contribute to tumor survival and development [9–12]. Cancer cells accelerate malignant progression by modulating glucose metabolism, with glycolysis serving as a major energy-producing pathway that is essential for tumor proliferation and metastasis [13–16]. For instance, NAT10 was found to promote glycolytic metabolism to facilitate cell growth, metastasis, as well as immunosuppression in CC by enhancing FOXP1 transcription [17]. Chen et al. reported that TRIM33 could boost glycolysis through increased ubiquitination with P53, thereby promoting esophageal cancer growth and progression [18].

Moreover, lactate, the final byproduct of glycolysis, serves a significant regulatory function in tumor progression [19–21]. Recently, it was uncovered that lactate-mediated lactylation of histone could regulate the expression of downstream targets, thereby promoting tumorigenesis, invasion and metastasis [22–25]. As revealed by Li et al., lactylation of H3K18la could confer immunotherapeutic resistance by promoting RUBCNL expression and activating cell autophagy in colorectal cancer [26]. In lung cancer, H3K18la lactylation level was strongly associated with worse clinical outcome and contributed to cellular immune escape through activation of POM121/MYC/ PD-L1 axis [27]. However, the underlying mechanisms by which CC cells modulate glycolytic metabolism as well as histone lactylation to facilitate tumor progression remain unclear.

Rab-interacting lysosomal protein-like 2 (RILPL2) belongs to the RILP family, which includes RILP, RILPL1, and RILPL2. This gene encodes a domain that resembles a rab-interacting lysosomal protein, suggesting its potential role in vesicular transport, the transport of cellular proteins, and the regulation of lysosomal morphology [28]. Recent research has indicated that RILPL2 is implicated in the biological functions of tumors associated with breast cancer and endometrial carcinoma [29, 30]. As revealed by Chen et al., RILPL2 suppressed breast cell growth, metastasis and enhanced chemotherapy sensitivity in breast cancer [29]. However, the functional role of RILPL2 in the CC has yet to be investigated.

In this work, we found that RILPL2 is a key target for CC and a valuable prognostic factor in CC. Our results uncovered a downregulation pattern of RILPL2 in CC specimens and cell lines. Overexpression of RILPL2 could restrain CC cell growth and glycolysis in vitro and inhibit tumorigenesis in vivo. Specifically, RILPL2 reduced LDHA protein stability by recruiting TRIM21, which in turn suppressed glycolytic metabolic reprogramming. In addition, by regulating glycolysis metabolism, RILPL2 decreased lactate production and blocked lactylation of H3K18la, thereby suppressing SOX9 and SMYD2 expression. Taken together, our results support an inhibitory effect of the RILPL2 on metabolic reprogramming and progression in CC and identify RILPL2 as a new potential therapeutic target.

## Materials and methods

### Cell culture

Human CC cell lines (HeLa, CaSki, and SiHa), human normal cervical epithelial cells (HcerEpic) and HEK293T cells were purchased from American Type Culture Collection (ATCC) (Manassas, VA). All cell lines were recently authenticated and tested for mycoplasma contamination. All cells were cultured in DMEM (12491015; Gibco) supplemented with 10% FBS (10099141C; Gibco) and 1% penicillin-streptomycin (C0222, Beyotime Biotechnology, Shanghai, China) and incubated with 5% CO_2_ at 37 °C.

### Cell viability analysis

Cell viability was detected by using a Cell Counting Kit-8 (CCK-8; Beyotime, China). Cells were seeded in 96-well plates at a density of 2000 cells per well. After cell attachment (0 h), absorbance was measured at 0, 24, 48, 72, and 96 h. At each time point, the culture medium was removed, and wells were gently washed with PBS. Then, 100 μL of CCK-8 working solution (prepared by diluting CCK-8 reagent 1:10 in serum-free medium) was added to each well, followed by a 2-h incubation at 37 °C. Absorbance was measured at 450 nm using a multifunctional microplate reader.

### Colony formation assay

Cells were seeded into six-well plates (200 cells per well) with DMEM supplemented with 10% FBS for approximately 14 days. When visible colonies formed, cells were fixed with methanol and stained with 0.1% crystal violet. Colonies were counted manually and imaged using a scanner.

### EdU assay

Cells were plated in 96-well plates at a density of 5000 cells per well with DMEM supplemented with 10% FBS for 24 h. Cells were then incubated with 50 μM EdU for 2 h, followed by fixation with 4% paraformaldehyde, permeabilization with 0.5% Triton X-100, and staining with Apollo reagent for 30 min. Nuclei were counterstained with Hoechst 33342.

### Flow-cytometric assay

Apoptosis was assessed using an apoptosis detection kit (Vazyme, China) following the manufacturer’s instructions. Cells were digested, resuspended in 500 μl of binding buffer with 5 μl of annexin V-FITC solution and 5 μl of propidium iodide (PI), and stained at room temperature for 15 min in the dark. Flow cytometry (FACScan; BD Biosciences, USA) and FlowJo software (BD, USA) were used to analyze the cells.

### TUNEL staining assay

TUNEL staining kit was used (Beyotime, China) according to the manufacturer’s instructions. Cells were fixed with 4% paraformaldehyde, permeabilized with 0.3% Triton X-100, and incubated with TUNEL reaction mixture at 37 °C in the dark. After washing with PBS, samples were mounted with anti-fade medium and observed under a fluorescence microscope. Both adherent and suspension cells were processed using the same protocol.

### Extracellular acidification rate

Extracellular acidification rate (ECAR) was measured using the Glycolysis Stress Test kit (Agilent Technologies, 103020-100) on a Seahorse XFe24 Extracellular Flux Analyzer (Agilent Technologies), according to the manufacturer’s instructions. Cells were washed and incubated in assay medium supplemented with 2 mM glutamine at 37 °C in a non-CO₂ incubator for 30 min. ECAR was recorded following the sequential injection of glucose (10 mM), oligomycin (1.5 μM), and 2-deoxy-D-glucose (50 mM).

### Measurement of lactate production

Cellular lactate levels were measured using an l-Lactate Assay Kit (Abcam, ab65330) according to the manufacturer’s instructions. Briefly, tissues were homogenized in lysis buffer on ice, and the lysates were centrifuged at 12,000×*g* for 10 min at 4 °C. The supernatants were collected for lactate quantification.

### Animal experiments

The Institutional Animal Care and Use Committee of Nanjing Medical University approved our protocol for all animal work. Five-week-old nude mice (BALB/c nude female mice) were purchased from GemPharmatech (Guangzhou, China) and raised under pathogen-free conditions. The nude mice were randomly divided into several groups according to experimental requirements (*n* = 6). The nude mice were injected subcutaneously and stably with transfected cells (2 × 10^6^) in 100 μL PBS.

### Clinical data and samples

The CC tissue microarray (HUteS120Su01) was acquired from Shanghai Outdo Biotech Co., Ltd. with 105 CC tissues and 15 adjacent tissues. The clinical data of patients is shown in Table S1. Moreover, CC tissues and adjacent non-tumor normal tissues of the in-house cohort were collected during surgery. All tissue specimens were quickly frozen in liquid nitrogen until RNA and protein extraction. This study was approved by the Institutional Review Board and the First Affiliated Hospital of Nanjing Medical University Ethics Committee, and informed consent was obtained from all patients. The clinical information of the patients is shown in Table S2.

### Statistical analysis

The data were shown as the mean ± SD of at least three independent experiments. The statistical analysis was carried out with GraphPad Prism 10 (GraphPad Software, USA). Student’s *t*-test was used for comparisons between two groups, and one-way or two-way ANOVA followed by Tukey’s post hoc test for multiple comparison. Survival analysis was performed using the log-rank test. Significance in the figures was indicated as follows: ns for *p* > 0.05; *p < 0.05; **p < 0.01; and ***p < 0.001.

Additional methodological details are described in the Supplementary Materials and Methods.

## Results

### RILPL2 is downregulated in CC and associated with better prognosis

**Fig. 1:**
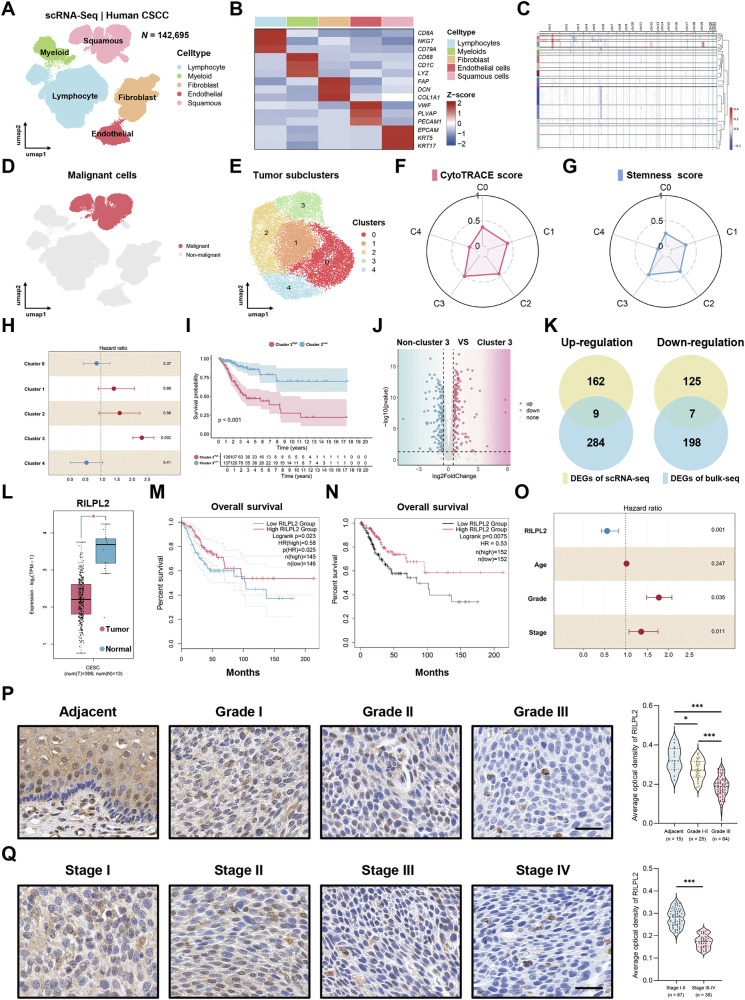
RILPL2 is lowly expressed in CC and correlated with a better prognosis. **A** Overall UMAP plot of 142,695 cells showing the five major cell populations. **B** Heatmap displaying cell markers in each of the major cell types. **C** The landscape of inferred copy-number variations (CNVs) for all squamous cells. **D** Malignant cells were identified with a probability score >0.99 by CNVs analysis. **E** The UMAP plot showing tumor cell clusters. **F**, **G** The radar plots displaying CytoTRACE score and stemness score across five tumor subclusters. **H** Hazard ratio forest plot comparing survival risk among five tumor subclusters. **I** Kaplan–Meier analysis (log-rank test) of the overall survival for tumor cluster 3. **J** The volcano plot of differentially expressed genes (DEGs) in tumor cluster 3. **K** Venn diagrams summarizing DEGs identified in scRNA-seq and bulk RNA-seq analyses. **L** The expression of RILPL2 in tumor tissues and normal tissues from the GEPIA2 database. **M**, **N** Overall survival analysis (log-rank test) of RILPL2 from the GEPIA2 database and Kaplan–Meier plotter database. **O** The independent prognostic value of RILPL2 was confirmed by multivariate Cox analysis. **P** Representative images and statistical graph (*P < 0.05, ***P < 0.001, one-way ANOVA with post hoc test) of IHC staining analysis of RILPL2 protein levels with different pathological grades (adjacent, Grades I, II, and III). Scale bar, 100 μm. **Q** Representative images and statistical graph (***P < 0.001, unpaired two-tailed Student’s *t*-test) of IHC staining analysis of RILPL2 protein levels at different stages (7th edition of the AJCC: Stage I, II, III, and IV). Scale bar, 100 μm.

We first performed single-cell analysis from a scRNA-seq dataset (HRA004971) of CC. Following quality filtering and cell annotation, we obtained five main cell types based on specific markers, including lymphocyte, myeloid, fibroblast, endothelial cell, and squamous cell (Fig. 1A, B and Table S3). Then, CNV analysis was conducted to determine malignant cells in CC with a CNV probability score >0.99 (Fig. 1C, D). To decipher tumor heterogeneity in CC, we extracted all malignant cells and employed subcluster analysis to identify five different subpopulations (Fig. 1E and Table S4). CytoTRACE is an emerging algorithm which can be used to infer the malignant potential of tumor subpopulations. It assesses the state of cell differentiation based on transcriptional diversity, which in turn evaluates the cell malignant potential. The results showed significant differences in CytoTRACE scores across the five identified cell clusters (clusters 0 to 4). Among these, cluster 3 exhibited the highest CytoTRACE scores, indicating a more undifferentiated and potentially more malignant state compared to the other clusters (Fig. 1F). Consistent with the CytoTRACE results, we found that cluster 3 had a relatively high Stemness score, further supporting its high degree of malignancy (Fig. 1G). To investigate the prognostic value of five subclusters, a deconvolution algorithm was employed to estimate the proportion of each cluster in the TCGA-CESC cohort. Using this approach, we quantified the relative abundance of five subclusters in individual patient samples and assessed their prognostic value. Cox regression revealed that cluster 3 was greatly associated with a poor prognosis, with a hazard ratio (HR) of 2.5 (Fig. 1H). Moreover, survival analysis demonstrated that patients with a higher proportion of cluster 3 cells had remarkably poor clinical outcome compared to those with lower proportions (Fig. 1I). To detect the molecular characteristics of cluster 3, a total of 303 DEGs were obtained by differential gene expression analysis (*P* < 0.05, |log2 FC| > 0.5) by comparing cluster 3 to all other clusters (non-cluster 3) (Fig. 1J and Table S5). Then, we intersected the DEGs identified from cluster 3 in the single-cell analysis with DEGs derived from bulk RNA-seq data of TCGA-CESC, resulting in 16 overlapping genes (Fig. 1K and Tables S6 and S7). Univariate Cox analysis showed that three genes were remarkably associated with prognosis in CC (Supplementary Table S8).

Given the limited research on RILPL2 in CC, we selected it for further analysis. It was shown that RILPL2 was lowly expressed in tumor specimens compared with normal samples from the TCGA-CESC cohort (Fig. 1L). Based on the GEPIA database, the survival curve revealed that high RILPL2 expression in CC had a better prognosis (Fig. 1M). Similar results were also observed in another cohort from the Kaplan–Meier Plotter website (Fig. 1N). The independent prognostic value of RILPL2 in CC was demonstrated by multivariate Cox analysis (Fig. 1O). Subsequently, we detected the clinical relevance of RILPL2 in CC. It was demonstrated that the RILPL2 protein level was greatly lower in CC tissues than in adjacent tissues by both Immunohistochemical and Western blot assays (Fig. S1A, B). Furthermore, we tried to uncover the relationship between RILPL2 protein levels and clinicopathological features of CC. RILPL2 expression showed a negative correlation with the Grade and Stage of CC (Fig. 1P, Q). Also, similar results were observed by Western blot analysis in CC tissues (Fig. S2A, B).

### RILPL2 inhibits the proliferation and induces apoptosis of CC cells

**Fig. 2:**
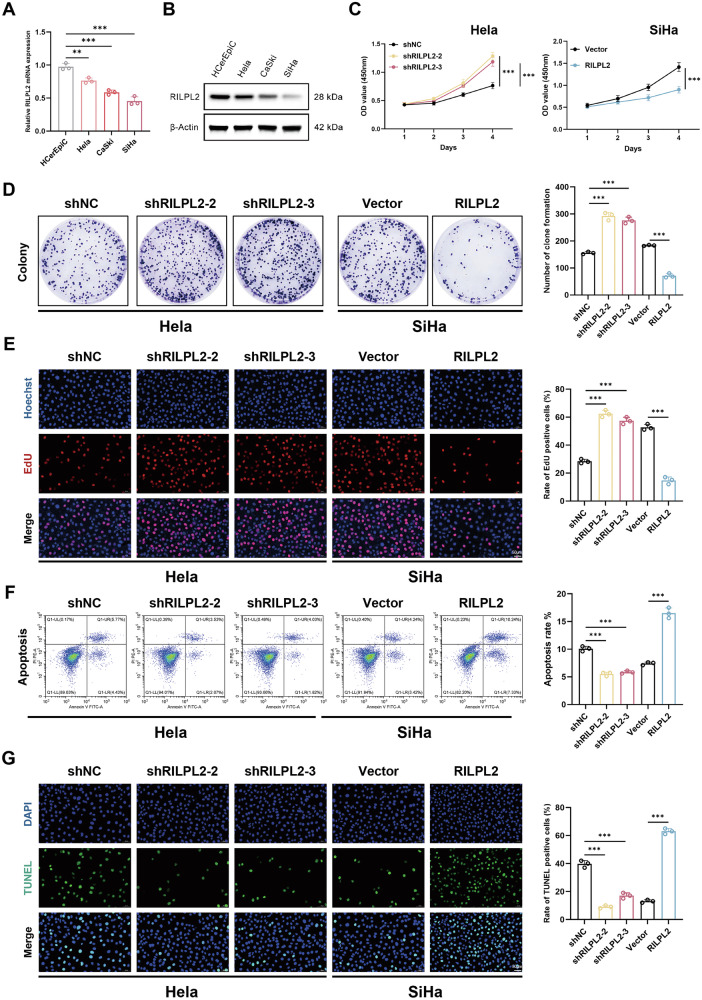
RILPL2 inhibits proliferation and induces apoptosis of CC cells. **A**, **B** The expression pattern of RILPL2 in CC cell lines was detected by qRT-PCR (*n* = 3 independent experiments, **P < 0.01, ***P < 0.001, one-way ANOVA with post hoc test) and Western blot. The effect of RILPL2 on proliferation in CC cells using **C** CCK-8 (*n* = 3 independent experiments, ***P < 0.001, two-way ANOVA with post hoc test), **D** colony formation, and **E** EdU assay (*n* = 3 independent experiments, ***P < 0.001, one-way ANOVA with post hoc test). Scale bar, 50 μm. The effect of RILPL2 on cell apoptosis was evaluated by **F** flow-cytometric analysis and **G** TUNEL assay (*n* = 3 independent experiments, ***P < 0.001, one-way ANOVA with post hoc test). Scale bar, 50 μm.

Next, we confirmed that RILPL2 was downregulated in CC cell lines by qRT-PCR and Western blot (Fig. 2A, B). Based on the expression pattern of RILPL2 in CC cell lines, HeLa cells with high expression of RILPL2 were chosen to knockdown RILPL2 through transfection of shRILPL2. Additionally, RILPL2 was stably overexpressed in low RILPL2-expression SiHa cells. Fig. S3 displays the favorable transfection efficiency in the indicated cell lines. CCK-8 assays revealed that silencing RILPL2 facilitated cell viability in HeLa cells, whereas overexpression of RILPL2 suppressed cell viability in SiHa cells (Fig. 2C). Moreover, both clonogenic formation and EdU assays confirmed that cell growth was remarkably boosted in HeLa cells following RILPL2 knockdown, whereas it was suppressed in SiHa cells when RILPL2 was overexpressed (Fig. 2D, E). Furthermore, the flow-cytometric analysis revealed that knockdown of RILPL2 led to a reduction in apoptosis in HeLa cells, whereas the overexpression of RILPL2 contributed to an increase in cell apoptosis in SiHa cells (Fig. 2F). Also, similar results were observed by TUNEL assay (Fig. 2G). To further confirm the functional role of RILPL2, rescue experiments were performed by re-expressing RILPL2 in RILPL2-silenced HeLa cells. The results revealed that RILPL2 silencing greatly facilitated cell proliferation and inhibited apoptosis, whereas restoration of RILPL2 expression markedly reversed these effects (Figs. S4 and S5). Collectively, the above results showed that RILPL2 inhibited CC proliferation and induced apoptosis in vitro.

### RILPL2 modulates metabolic reprogramming to suppress CC cells' survival

**Fig. 3:**
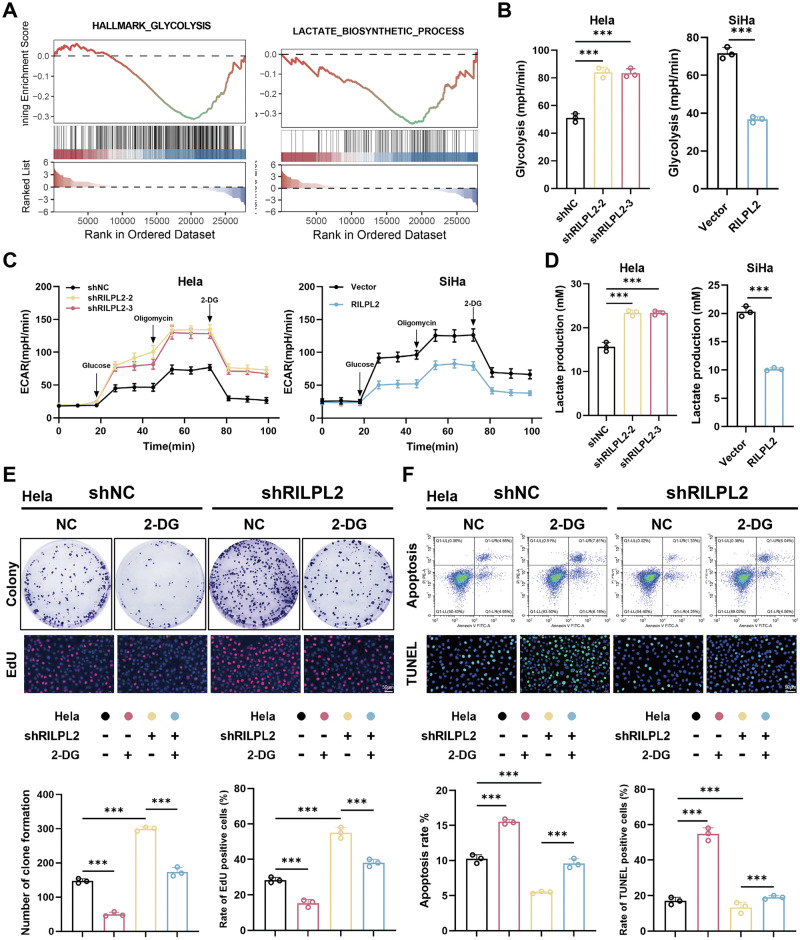
RILPL2 modulates metabolic reprogramming to suppress CC cells' survival. **A** GSEA analysis of the TCGA dataset indicated the glycolysis pathway and lactate synthesis process were inhibited after RILPL2 overexpression. The effect of RILPL2 on glycolysis in vitro was measured by **B** glycolytic activity assay (*n* = 3 independent experiments, ***P < 0.001, one-way ANOVA with post hoc test and unpaired two-tailed Student’s *t*-test), **C** extracellular acidification rate (ECAR) analysis, and **D** lactate production assay (*n* = 3 independent experiments, ***P < 0.001, one-way ANOVA with post hoc test and unpaired two-tailed Student’s *t*-test). Cell proliferation was detected using **E** colony formation and EdU assay (*n* = 3 independent experiments, ***P < 0.001, one-way ANOVA with post hoc test) in different experimental groups. Scale bar, 50 μm. Cell apoptosis was examined using **F** flow-cytometric analysis and TUNEL assay (*n* = 3 independent experiments, ***P < 0.001, one-way ANOVA with post hoc test) in different experimental groups. Scale bar, 50 μm.

To investigate the mechanism of the inhibitory effect of RILPL2, GSEA analysis was employed using the TCGA-CESC dataset based on RILPL2 transcriptional levels. It was shown that glycolysis as well as lactate biosynthetic pathways were blocked in the RILPL2 high expression group, suggesting RILPL2 might inhibit CC development by attenuating metabolic reprogramming (Fig. 3A). As expected, both glycolytic activity and ECAR assays indicated that glycolysis was greatly boosted in Hela cells following RILPL2 knockdown, whereas it was inhibited in SiHa cells when RILPL2 was overexpressed (Fig. 3B, C). Moreover, silencing RILPL2 notably enhanced lactate levels in CC cells, while overexpression of RILPL2 contributed to a significant decrease in lactate levels (Fig. 3D). Subsequently, 2-DG (a glycolysis inhibitor) was used to detect whether RILPL2 exerts its role in a glycolysis-dependent way. As depicted in Fig. 3E, F, 2-DG reversed the oncogenic effect of RILPL2 knockdown in HeLa cells. Moreover, rescue experiments indicated that restoration of RILPL2 expression greatly reversed the enhanced glycolysis process induced by knockdown of RILPL2 in HeLa cells (Fig. S6). Therefore, these results confirmed that RILPL2 suppressed CC progression via inhibiting the glycolysis pathway.

### RILPL2 inhibits glycolysis in CC cells via interacting with LDHA

**Fig. 4:**
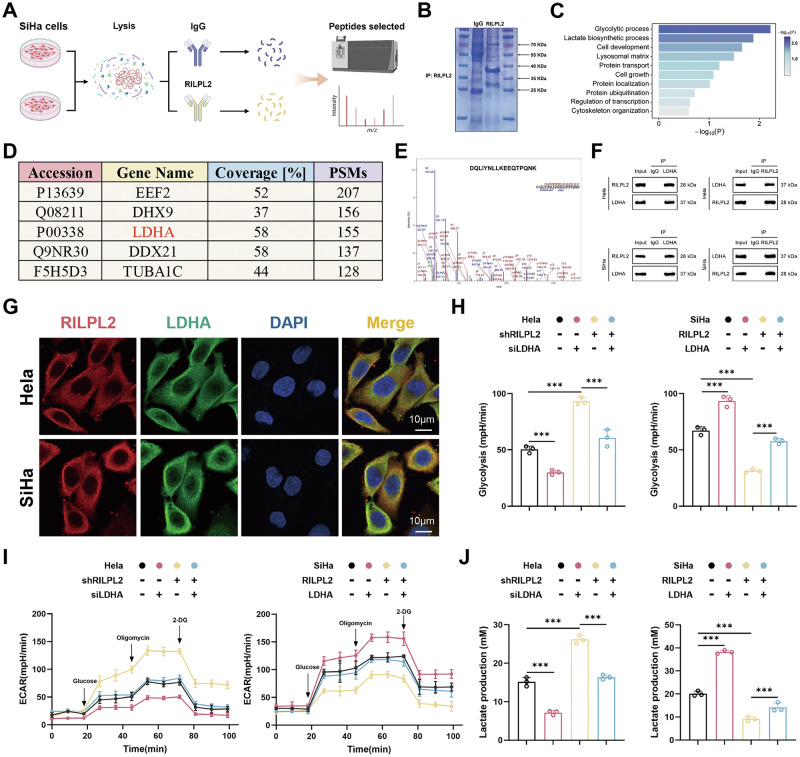
RILPL2 inhibits glycolysis in CC cells via interacting with LDHA. **A** Scheme for the identification of potential proteins interacting with RILPL2 by immunoprecipitation-mass spectrometry (IP-MS). **B** Coomassie blue staining of IP cell lysates. **C** Functional enrichment analysis of RILPL2 interactors. **D** List of top five potential proteins identified by IP/MS analysis. **E** IP/MS analysis revealed that LDHA interacts with RILPL2. **F** Examination of endogenous protein interactions between RILPL2 and LDHA in CC cells lysates. **G** Immunofluorescence assay demonstrated the co-localization of RILPL2 and LDHA. Scale bar, 10 μm. Cell glycolysis was detected using **H** glycolytic activity assay (*n* = 3 independent experiments, ***P < 0.001, one-way ANOVA with post hoc test), **I** ECAR analysis and **J** lactate production assay (*n* = 3 independent experiments, ***P < 0.001, one-way ANOVA with post hoc test) in different experimental groups.

To determine the underlying molecular mechanism of RILPL2 in CC, IP/MS was applied in SiHa cells (Fig. 4A, B). Function and pathway enrichment analysis of top candidate proteins indicated that the glycolysis process was highly enriched (Fig. 4C). Lactate dehydrogenase A (LDHA), as a previously reported critical player in the glycolytic pathway, was selected for the next experiments with a high IP/MS PSMs score (Fig. 4D, E and Table S13). Co-IP analysis also verified the direct interaction between RILPL2 and LDHA (Fig. 4F). Immunofluorescence assays demonstrated the co-localization of RILPL2 and LDHA in CC cells (Fig. 4G). Next, it was demonstrated that silencing LDHA counteracted the promotive role of RILPL2 knockdown on glycolysis in HeLa cells by both glycolytic activity and ECAR assays (Fig. 4H, I). Moreover, the increased lactate production in RILPL2 knockdown cells was rescued via the downregulation of LDHA (Fig. 4J). In contrast, opposite results were observed in SiHa cells under simultaneous treatment with RILPL2 overexpression and LDHA overexpression vectors (Fig. 4H–J).

### RILPL2 restrains the CC cells' survival via LDHA

**Fig. 5:**
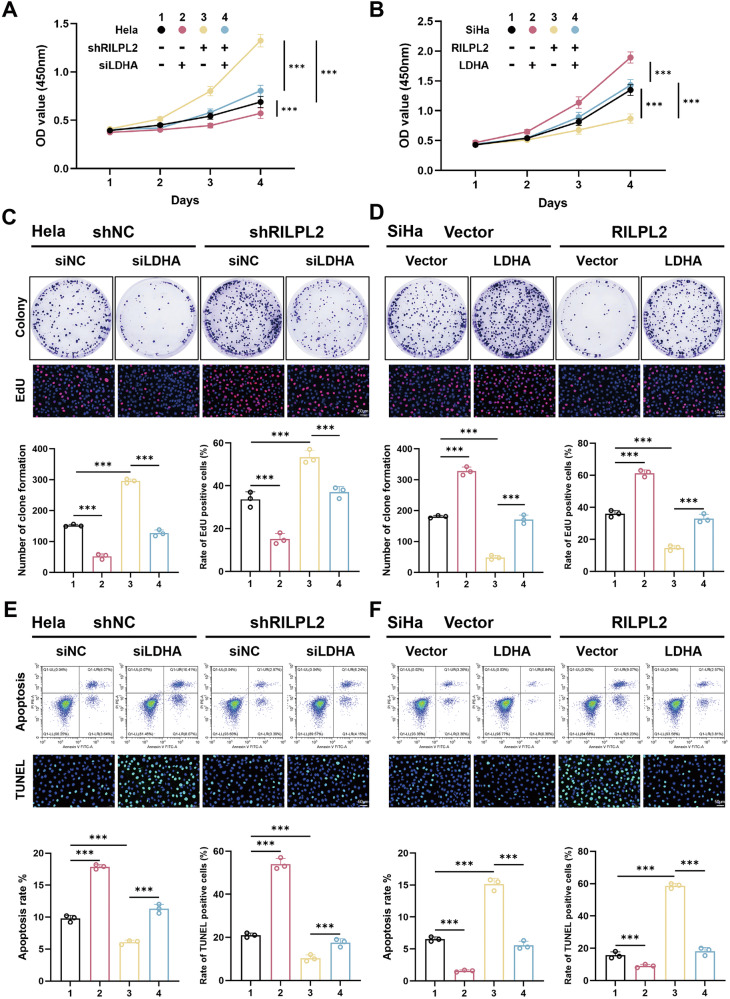
RILPL2 restrains the CC cells' survival via LDHA. **A**, **B** CCK-8 (*n* = 3 independent experiments, ***P < 0.001, two-way ANOVA with post hoc test), **C**, **D** colony formation, and EdU assays (*n* = 3 independent experiments, ***P < 0.001, one-way ANOVA with post hoc test) were employed to detect cell proliferation in different experimental groups. Scale bar, 50 μm. **E**, **F** Flow-cytometric analysis and TUNEL assay (*n* = 3 independent experiments, ***P < 0.001, one-way ANOVA with post hoc test) were conducted to examine cell apoptosis in different experimental groups. Scale bar, 50 μm.

We further explored whether RILPL2 regulates CC cell proliferation and apoptosis through LDHA via rescue experiments. CCK-8 experiments demonstrated that downregulation of LDHA attenuated the increase in cell viability induced by silencing RILPL2 (Fig. 5A, B). The results of colony formation and EdU experiments confirmed that the increased cell proliferation in RILPL2-silenced cells was counteracted after silencing LDHA, whereas LDHA overexpression diminished the decreased cell growth in RILPL2-upregulated cells (Fig. 5C, D). Conversely, silencing LDHA abolished inhibition in apoptosis by downregulation of RILPL2 in HeLa cells, while upregulation of LDHA in SiHa cells greatly reversed apoptosis induced by RILPL2 overexpression (Fig. 5E, F).

### RILPL2 facilitates LDHA degradation through TRIM21-mediated ubiquitination

**Fig. 6:**
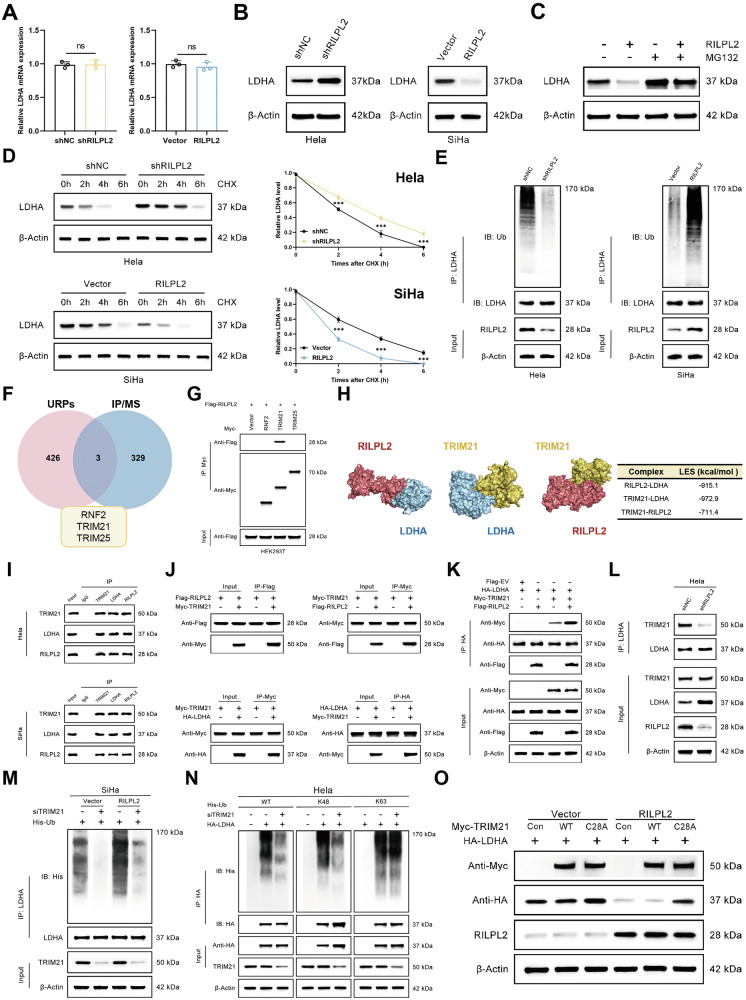
RILPL2 facilitates LDHA degradation through TRIM21-mediated ubiquitination. **A** Detecting LDHA mRNA levels in CC cells by qRT-PCR in the indicated groups. **B** Detecting LDHA protein levels in CC cells by Western blot in the indicated groups. **C** Assessment of LDHA protein levels in overexpressed RILPL2 CC cells with or without proteasome inhibitor MG132 treatment. **D** Representative images and the corresponding quantification of the Western blot analysis (*n* = 3 independent experiments, ***P < 0.001, two-way ANOVA with post hoc test) were conducted to assess the half-life of the LDHA protein in CC cell lines. **E** Assessment of endogenous LDHA ubiquitination in CC cells transfected with the indicated groups. **F** Potential proteins were screened through the intersection of URPs from the Genecard database and IP/MS analysis. **G** Co-IP assay on HEK293T cells transfected with Myc-tagged RNA2, TRIM21, and TRIM25 plasmids. **H** Predicted binding complex and the lowest energy structure (LES) of RILPL2, TRIM21, and LDHA by molecular docking analysis. **I** Detection of endogenous protein interactions between RILPL2, TRIM21, and LDHA in CC cells lysates. **J** Detection of exogenous protein interactions between RILPL2, TRIM21, and LDHA in 293T cells. Flag-RILPL2 and Myc-TRIM21, and HA-LDHA plasmids were transfected into HEK293T cells. **K** Western blot detection of Flag, HA, and Myc- tagged proteins after IP in cells co-transfected with Flag-RILPL2 and Myc-TRIM21. **L** Assessment of endogenous Co-IP of LDHA and TRIM21 in shNC or shRILPL2. **M** Ubiquitination level of LDHA in SiHa-Vector/SiHa-RILPL2 cells were transiently transfected with TRIM21 siRNA. **N** Ubiquitination level of HA-LDHA in siTRIM21 and corresponding control HeLa cells. **O** SiHa-Vector/SiHa-RILPL2 cells were transiently transfected with Myc-TRIM21-Con/WT/C28A and HA- LDHA plasmids.

To further explore the molecular mechanism of the interaction between RILPL2 and LDHA, we first detected if RILPL2 regulates LDHA mRNA or protein levels by qRT-PCR analysis and Western blotting. Interestingly, alteration of RILPL2 expression greatly changed LDHA protein levels but had no effect on LDHA mRNA levels. (Fig. 6A, B). Moreover, treatment with the proteasome inhibitor MG132 counteracted the reduction in LDHA protein levels after upregulation of RILPL2. (Fig. 6C). We then investigated the role of RILPL2 on endogenous LDHA protein stability after treatment with the protein synthesis inhibitor cycloheximide (CHX). The results revealed that knockdown of RILPL2 remarkably inhibited LDHA degradation, while upregulation of RILPL2 notably enhanced LDHA degradation (Fig. 6D). Subsequently, we detected the effect of RILPL2 on the ubiquitination of endogenous LDHA in CC cells. It was observed that silencing RILPL2 greatly decreased LDHA ubiquitination in HeLa cells, whereas overexpression of RILPL2 remarkably increased ubiquitination of LDHA in SiHa cells (Fig. 6E). In addition, re-expression of RILPL2 abolished the increased LDHA protein expression and stability induced by RILPL2 knockdown in HeLa cells, and rescued LDHA ubiquitination levels (Fig. S7A–D).

The above findings from IP/MS suggested the presence of proteins that mediate ubiquitination among the proteins interacting with RILPL2 (Fig. 4B). Considering that RILPL2 is not a ubiquitinating enzyme, we thus speculate that RILPL2 may mediate the ubiquitination of LDHA by recruiting ubiquitination-related proteins (URPs). To identify the potential URPs, the intersection was made between the proteins binding to RILPL2 from IP-MS results and the URPs list from the Genecard database (Table S14), resulting in three shared proteins (RNF2, TRIM21, and TRIM25) (Fig. 6F). To identify potential URPs binding to RILPL2, we co-transfected Flag-tagged RILPL2 (WT) with Myc- RNF2, TRIM21, and TRIM25 into HEK293T cells. Co-IP experiments indicated that RILPL2 interacted only with TRIM21, but not with RNF2 or TRIM25 (Fig. 6G). Subsequently, molecular docking was conducted to verify the interaction pattern of RILPL2, TRIM21 and LDHA with the lowest energy structure (LES) score (Fig. 6H). Co-IP analysis demonstrated the presence of both RILPL2 and TRIM21 in protein precipitates labeled with LDHA, while LDHA and TRIM21 were identified within protein precipitates tagged with RILPL2 in CC and 293T cell lines (Fig. 6I, J). After co-transfection of Flag-RILPL2 and Myc-TRIM21, we observed that in the absence of TRIM21, RILPL2 could bind to LDHA (Fig. 6K lane 2); in the absence of RILPL2, TRIM21 could also bind to LDHA (Fig. 6K, lane 3); and when Myc-TRIM21 was transfected at the same time, TRIM21 bound to LDHA was significantly increased upon RILPL2-Myc (Fig. 6K, lane 4). In addition, endogenous Co-IP was performed in HeLa cells with knockdown of RILPL2, demonstrating that TRIM21 binding to LDHA was reduced with the decline of RILPL2 (Fig. 6L). Rescue experiments demonstrated that re-introduction of RILPL2 could restore the interaction between TRIM21 and LDHA in RILPL2-silenced HeLa cells (Fig. S7E).

Transient transfection of SiHa cells with TRIM21 siRNA demonstrated that silencing TRIM21 significantly reduced the ubiquitination level of LDHA (Fig. 6M). Further results indicated that silencing TRIM21 dramatically reduced the K48-linked but not the K63-linked polyubiquitination of LDHA (Fig. 6N). To detect whether RILPL2-mediated LDHA degradation is dependent on the ubiquitinase activity of TRIM21, the C28A mutant of TRIM21 was constructed and transfected into Vector/RILPL2 SiHa cells. The results showed that LDHA protein was more stable in the TRIM21-C28A group, whereas it was markedly diminished in the TRIM21-WT group, and this change was more obvious in the RILPL2 overexpression group (Fig. 6O).

Furthermore, we confirmed the molecular mechanism of RILPL2 in normal cells (HEK293T). Compared with CC cell lines (HeLa and SiHa), RILPL2 expression levels were higher in HEK293T cells (Fig. S8A). As shown in Fig. S8B–D, silencing RILPL2 remarkably suppressed LDHA degradation and increased LDHA protein level through inhibition of LDHA ubiquitination in HEK293T cells. Moreover, the regulation of LDHA ubiquitination by RILPL2 was dependent on its promotion of the interaction between TRIM21 and LDHA in HEK293T cells (Fig. S8E, F). Collectively, our findings support the role of RILPL2 in degrading LDHA proteins via TRIM21-mediated ubiquitination of K48 from LDHA.

### RILPL2 blocks H3K18 lactylation in CC cells via LDHA

**Fig. 7:**
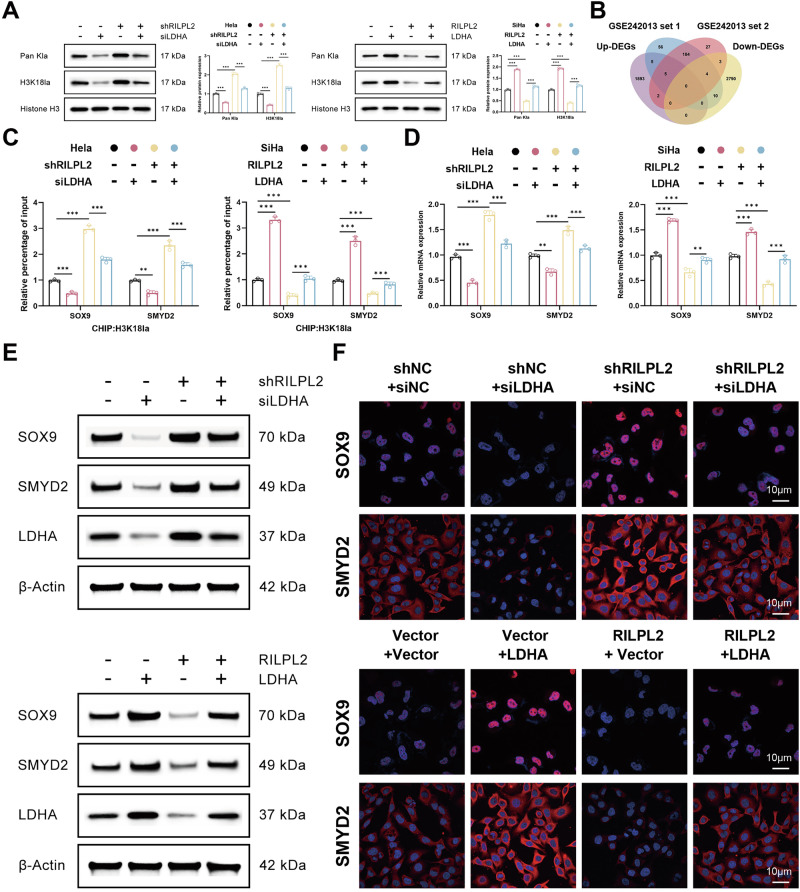
RILPL2 blocks H3K18 lactylation in CC cells via LDHA. **A** The levels of pan histone and H3K18 site lactylation were detected by western blot analysis (*n* = 3 independent experiments, ***P < 0.001, one-way ANOVA with post hoc test) in different experimental groups. **B** RILPL2 -related target genes were identified through the intersection of RNA-seq and GSE242013. **C** The H3K18la enrichment in SOX9 and SMYD2 was measured by ChIP-qPCR assay (*n* = 3 independent experiments, **P < 0.01, ***P < 0.001, one-way ANOVA with post hoc test) in different experimental groups. The expression level of SOX9 and SMYD2 was examined by **D** qRT-PCR (*n* = 3 independent experiments, **P < 0.01, ***P < 0.001, one-way ANOVA with post hoc test) and **E** Western blot in different experimental groups. **F** Representative immunofluorescence images of SOX9 and SMYD2 in different experimental groups.

Lactate-induced histone lactylation has recently been identified as a distinct epigenetic mechanism involved in the regulation of gene transcription and expression [31]. Considering our previous data demonstrating the role of RP2 in regulating lactate production, we further detected the effect of RILPL2 on pan histone lactylation and specifically on H3K18 lactylation (H3K18la) in CC cells. It was observed that overexpression of RILPL2 inhibited pan histone and H3K18 lactylation in CC cells, whereas pan histone and H3K18la were greatly facilitated after RILPL2 knockdown (Fig. S9A). Previous findings have demonstrated that higher expression levels of LDHA enhanced pan histone lactylation and H3K18la in tumor cells [32–34]. It was uncovered that overexpression of LDHA inhibited the inhibitory effect of RILPL2 overexpression on pan histone lactylation and H3K18la (Fig. 7A).

To determine the underlying target genes of H3K18la induced by knockdown of RILPL2, we first performed RNA-seq on HeLa cells with shRILPL2 as well as corresponding wild-type cells and obtained 1905 up-DEGs and 2809 down-DEGs (Table S15). After intersection analysis with an H3K18la chip-seq dataset (GSE242013, Table S16), a total of 12 overlapping genes remained. Among these shared genes, SOX9 and SMYD2, as oncogenes, were shown to be involved in regulating CC progression in previous studies and were selected for subsequent experiments [35–37]. Next, a ChIP-qPCR assay was applied to examine the enrichment of H3K18la signals of all tested genes. It was shown that silencing RILPL2 increased H3K18la enrichment of SOX9 and SMYD2. In contrast, overexpression of RILPL2 led to a significant reduction in H3K18la levels of SOX9 and SMYD2 (Fig. S9B). As expected, the increased expression of SOX9 and SMYD2 was found in the RILPL2 overexpression group, while RILPL2 knockdown inhibited the expression of these two genes by qRT-PCR and Western blot assays (Fig. S9C, D). Further funding from the ChIP-qPCR assay revealed that upregulation of LDHA reversed the suppression of SOX9 and SMYD2 binding to H3K18la in CC cells induced by overexpression of RILPL2 (Fig. 7C). Additionally, Fig. 7D, E indicated that upregulation of LDHA rescued the reduced expression of SOX9 and SMYD2 due to upregulation of RILPL2. Meanwhile, similar results were obtained by IF assays in CC cells (Fig. 7F). In summary, our findings indicated that RILPL2 overexpression ultimately restrained CC cells' progression by suppressing LDHA-mediated cellular glycolysis, reducing lactate production, weakening H3K18la, and inhibiting the transcription of SOX9 and SMYD2.

### RILPL2 inhibits CC cell growth in vivo and is negatively correlated in clinical CC samples

To investigate the effect of RILPL2 on CC tumorigenesis in vivo, transfected HeLa and SiHa cells were subcutaneously injected into nude mice to generate xenograft models.

**Fig. 8:**
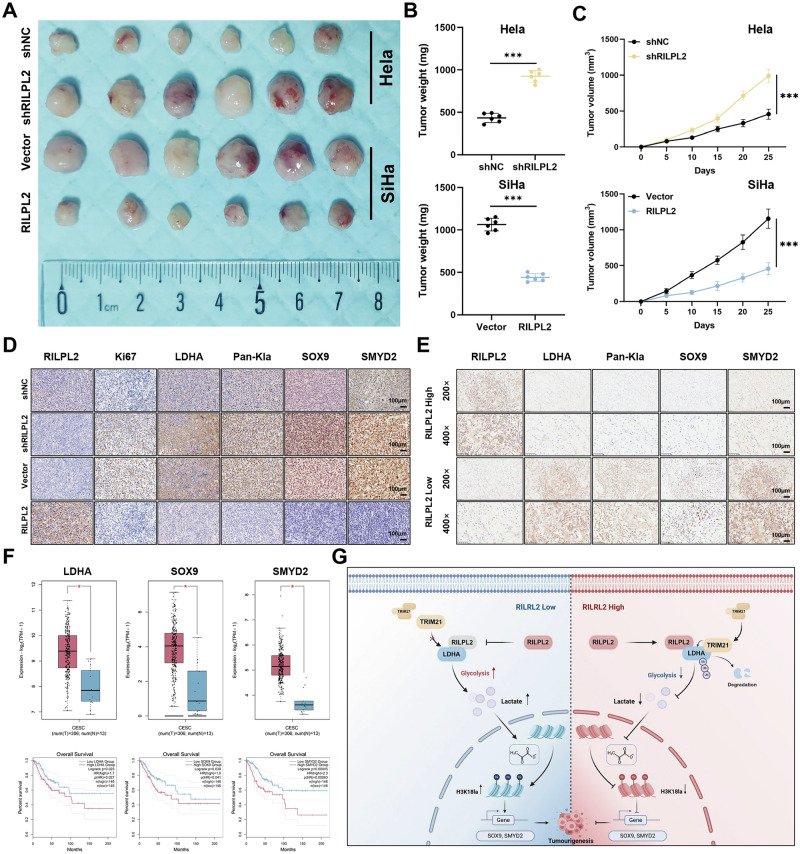
RILPL2 inhibits CC cell growth in vivo and is negatively correlated in clinical CC samples. **A** Representative images of subcutaneous tumors of CC cells with RILPL2 knockdown or overexpression. **B**, **C** Tumor weight (*n* = 6, ***P < 0.001, unpaired two-tailed Student’s *t*-test) and time course of growth (*n* = 6, ***P < 0.001, two-way ANOVA with post hoc test) of CC xenografts. **D** RILPL2, Ki67, LDHA, Pan Kla, SOX9, and SMYD2 expression was measured in tumors by IHC staining. Scale bars: 100 μm. **E** Representative images of IHC staining of RILPL2, Ki67, LDHA, Pan Kla, SOX9, and SMYD2 in clinical specimens. Scale bars: 100 μm. **F** Expression pattern and survival analyses for LDHA, SOX9, and SMYD2 based on the GEPIA2 database. **G** The mechanistic scheme of this study.

It was shown that the volume and weight of tumors in the RILPL2 knockdown group were markedly elevated when compared to the negative control group. Conversely, the overexpression of RILPL2 was associated with a decrease in the growth, size, and weight of xenografted tumors in vivo (Fig. 8A–C). IHC staining for xenografted tumors demonstrated a lower protein level of Ki67, LDHA, Pan Kla, SOX9, and SMYD2 in the RILPL2 overexpression group (Fig. 8D). Next, we found that RILPL2 protein level was negatively correlated with that of LDHA, Pan Kla, SOX9, and SMYD2 in CC clinical specimens (Fig. 8E). Further analyses indicated that LDHA, SOX9, and SMYD2 were highly expressed in the CC tumor group and were correlated with poor clinical outcome in patients with CC (Fig. 8F).

## Discussion

Through a combination of function experiments conducted both in vitro and in vivo, the current study highlights the functional role of RILPL2 as a significant inhibitor of CC progression and development. We observed that RILPL2 presented a lower expression in CC tissues and was associated with a favorable prognosis. Several cellular experiments were conducted to detect the effect of RILPL2 on cell growth, apoptosis, and glycolytic activity in CC. It was shown that RILPL2 could suppress malignant phenotype via TRIM21-mediated K48 ubiquitination of LDHA to promote the protein degradation of LDHA. Various rescue experiments were employed to verify the RILPL2/ LDHA axis in the suppression of glycolysis, lactate production and progression of CC. By regulation of metabolic reprogramming, it was further found that RILPL2 could abolish lactylation of H3K18la in an LDHA-dependent way, contributing to the downregulation of the SOX9 and SMYD2 (Fig. 8G).

Numerous reports have highlighted the significant function of RILPL2 in different types of tumors [29, 30, 38]. In breast cancer, RILPL2 inhibits tumor growth by binding to TUBB3 and regulating the PTEN/AKT pathway. It also reverses resistance to Taxotere-mediated apoptosis, highlighting its therapeutic significance [29]. As revealed by Jiang et al., RILPL2 was significantly downregulated in lung cancer and was associated with better clinical outcomes. RILPL2 was positively correlated with immune cell infiltration and PD-L1 expression, suggesting its involvement in tumor immunity and potential for predicting immunotherapy response [38]. In the present study, we observed a notable downregulation of RILPL2 in CC specimens and correlated with a favorable CC clinical outcome. Next, we confirmed the lower expression pattern of RILPL2 in CC cell lines. Interestingly, it was observed that RILPL2 expression was relatively high in HeLa cells but relatively low in SiHa cells, which might be attributed to cell line-specific epigenetic modification, transcriptional regulation, and post-translational modifications. We will further explore the molecular mechanisms that lead to this difference in the future. Moreover, a comprehensive set of experiments revealed that RILPL2 plays a critical role in suppressing tumor growth and glycolytic activity in CC. However, the underlying mechanisms through which RILPL2 exerts its inhibitory effects on the malignant characteristics of CC remain to be thoroughly explored.

Then, we applied IP/MS to unearth the potential mechanism involved in the inhibitory effects of RILPL2 on CC development. LDHA, an essential player in the glycolytic pathway [39–42], was determined as a potential protein binding to RILPL2 in CC cells. Our results further uncovered that upregulation of LDHA counteracted the inhibitory effect on cell viability and glycolytic activity induced by downregulation of RILPL2, indicating that RILPL2 inhibits CC progression through LDHA. Further data indicated that altering RILPL2 expression resulted in changes in LDHA protein expression, while LDHA mRNA levels were unaffected. This observation suggests that RILPL2 may play a role in the regulation of LDHA stability via post-translational modifications. To clarify the mechanism of RILPL2 in the regulation of LDHA protein stability, we blocked mRNA translation by treating CHX with CC cells, and the results showed that overexpression of RILPL2 reduced the protein level of LDHA even under the condition of translation blocking. Inhibition of proteasome activity using MG132 reversed the downregulation of LDHA protein induced by overexpression of RILPL2. Moreover, our data showed that the ubiquitination level of LDHA was greatly reduced in overexpressing RILPL2 CC cells. Consequently, we speculate that RILPL2 promotes the degradation of LDHA protein by increasing ubiquitination of LDHA protein, blocks the reprogramming of glycolytic metabolism and inhibits CC progression.

Further studies revealed that TRIM21 plays a crucial role in RILPL2-mediated LDHA degradation. As an E3 ubiquitin ligase, TRIM21 has been found to regulate the stability and degradation of key proteins in various cancers [43–45]. Accumulating studies have indicated that TRIM21 promotes the degradation of its target proteins through K48-linked ubiquitination, thereby modulating cell development and progression [46, 47]. Our Co-IP results confirmed the interaction between TRIM21 and RILPL2, and we further demonstrated that RILPL2 facilitates TRIM21-mediated K48-linked ubiquitination of LDHA, promoting its degradation and ultimately blocking glycolytic reprogramming to inhibit CC progression. This finding further supports the role of TRIM21 as a key ubiquitin ligase in metabolic regulation and suggests that RILPL2 may function as a crucial scaffold protein, recruiting TRIM21 to facilitate LDHA degradation and exert its tumor-suppressive function. This mechanism not only expands the understanding of the role of TRIM21 in the metabolic pathway but also provides new evidence for TRIM21 as a potential therapeutic target. Here, we uncovered a previously unrecognized molecular mechanism of RILPL2 in CC. Our findings demonstrated that RILPL2 functions as a critical molecular scaffold that recruits and facilitates the interaction between TRIM21 and the key glycolytic enzyme LDHA. This interaction markedly enhances the ubiquitination of LDHA, which in turn promotes its proteasomal degradation and reduces its protein stability. Moreover, the role of TRIM21 as an E3 ubiquitin ligase targeting LDHA in CC has not been previously described. The regulatory effect of RILPL2 on the TRIM21-LDHA axis further highlights the novelty and biological significance of this pathway. Given the central role of LDHA in aerobic glycolysis in tumor cells, these results provide evidence that RILPL2 suppresses tumor metabolism through post-translational regulation of glycolytic enzymes.

In addition to the direct effect on metabolic pathways, the influence of RILPL2 on novel epigenetic regulation (histone lactylation) further enhances the interplay between metabolism and gene expression in CC. In 2019, Zhao and his team made the groundbreaking discovery that lactate is an important material for histone lactylation [31]. This innovative post-translational modification plays an important role in activating gene expression by promoting H3K18 lactylation [27, 32, 48, 49]. In this study, to explore potential downstream target genes regulated by H3K18 lactylation (H3K18la) upon RILPL2 modulation, we integrated our RNA-seq data with a publicly available H3K18la ChIP-seq dataset (GSE242013). Notably, the ChIP-seq dataset was generated from ocular melanoma rather than CC, which represents a limitation of the current analysis, given that histone lactylation patterns may exhibit tissue-specific characteristics. Despite this limitation, the use of this ChIP-seq dataset provided a valuable reference for identifying genes potentially regulated by H3K18la, as histone lactylation is closely linked to cellular metabolic status, and may share conserved regulatory features across different tumor types. By intersecting our RNA-seq data and H3K18la chip-seq dataset, we identified SOX9 and SMYD2 as s overlapping downstream targets.

SOX9, a well-known transcription factor, plays a critical role in cell differentiation and tissue development [50, 51]. Numerous studies have uncovered upregulation of SOX9 across multiple tumor types, which correlates strongly with poor clinical outcomes [35, 52–54]. Moreover, SOX9 has been widely observed in promoting tumor progression, including CC, by boosting cell proliferation, stemness, metastasis, and metabolic process [55–57]. SMYD2 is a lysine methyltransferase originally identified for its role in histone H3K36 methylation modification [58, 59]. In addition to histones, SMYD2 also methylates various non-histone proteins, modulating their stability and biological activity [60–62]. Accumulating evidence suggested that SMYD2 acts as an oncogenic epigenetic regulator in diverse cancers by promoting cell cycle, metabolic reprogramming, as well as inhibiting apoptosis [63–65]. ChIP-qPCR revealed that upregulation of LDHA counteracted the inhibitory effect of upregulation of RILPL2 on H3K18la of SOX9 and SMYD2. Also, qRT-PCR and Western blot demonstrated that overexpression of LDHA was able to counteract the suppression of SOX9 and SMYD2 expression levels by upregulation of RILPL2. Our results suggested that RILPL2 inhibited glycolytic reprogramming of CC cells via LDHA, which reduced lactate production and suppressed H3K18la lactylation-mediated downstream gene expression, thereby restraining CC progression.

This study has several limitations. First, the single-cell RNA sequencing analyses were based on publicly available cervical cancer datasets rather than newly generated single-cell data from fresh clinical specimens. Future studies incorporating prospective single-cell sequencing analysis of tumor and adjacent tissues of CC will further refine the cellular heterogeneity and regulatory mechanisms identified here. Second, the tumor tissue microarray included an imbalanced number of tumor and adjacent non-tumor tissues. Although paired-sample analyses based on an in-house cohort were performed to support the conclusions, additional well-matched cohorts will be valuable for further validation.

## Conclusions

In conclusion, we demonstrate for the first time that RILPL2 was lowly expressed in CC and associated with better outcomes in CC patients. By recruiting TRIM21, which interacts with LDHA, RILPL2 facilitates the ubiquitination and degradation of LDHA protein, blocks metabolism reprogramming, which in turn inhibits CC development. Also, RILPL2/TRIM21/LDHA axis abolished H3K18la lactylation and downregulated SOX9 and SMYD2 related to malignant phenotype, contributing to inhibitory effect on CC cells. Therefore, the above results revealed that targeting the RILPL2/TRIM21/LDHA axis may be a promising therapeutic target for treating CC.

## Supplementary information

Supplementary Materials

Supplementary Tables

full uncropped Gels and Blots image(s)

Reproducibility Checklist

## Data Availability

The datasets used and/or analyzed during the current study are available from the corresponding author on reasonable request.
