# Supplementary material for: RILPL2 suppresses metabolic reprogramming and progression of cervical cancer by attenuating LDHA protein stability and inhibiting H3K18 lactylation
Source: Cell Death Dis. 2026 May 4;17(1):590. doi: 10.1038/s41419-026-08808-9 (PMC13284358; doi:10.1038/s41419-026-08808-9)
Supplement: Supplementary file 1 — Supplementary Materials [file 41419_2026_8808_MOESM1_ESM.docx]

**Supplementary Materials**

**Supplementary Materials and Methods**

**Bioinformatics analysis**

Single-cell analysis

A cervical cancer single-cell RNA sequencing (scRNA-seq) dataset (HRA004971) was collected from the National Genomics Data Center (https://ngdc.cncb.ac.cn/gsa-human/browse/HRA004971).

Seurat package in R (version 4.3.3, The R Foundation) was applied to perform quality controls and downstream analyses. ‘SeuratObject’ was created using the following parameter settings: min cells = 10 and min features = 100. Additionally, we filtered out low‐quality cells, defined as those with fewer than 200 unique molecular identifiers (UMIs), more than 6,000 UMIs, or mitochondrial gene expression exceeding the 25% threshold. As a result, 142,695 cells were generated for further analysis. Each sample was normalized with log transformation, and 2,000 highly variable features were determined by the ‘FindVariableFeatures’ function in Seurat with default parameters. All data were integrated using the ‘FindIntegrationAnchors’ function, defining anchor sets based on 2000 corresponding features between cells, which were selected using the ‘SelectIntegrationFeatures’ function.

After performing quality control, we scaled the expression of each gene and centered the data using the ‘ScaleData’ function. Principal component analysis (PCA) was performed to reduce data dimensionality using the ‘RunPCA’ function. We selected the first 30 principal components based on the elbow plot visualization. Next, Uniform Manifold Approximation and Projection (UMAP) was run using the ‘RunUMAP’ function to visualize the data in a lower‐dimensional space. We calculated the k‐nearest neighbors using the ‘FindNeighbors’ function and constructed a shared nearest neighbor (SNN) graph to identify the distinct groups of cells. Different cell types were identified for each cluster based on the expression of known marker genes.

Identification of Malignant Cells Using InferCNV

To distinguish malignant from non-malignant cells, we applied InferCNV (v1.10.1) to infer large-scale chromosomal copy number variations (CNVs) from single-cell RNA-seq data. Raw count matrices were used as input, with immune and stromal cell types (e.g., T cells, B cells, endothelial cells) designated as reference diploid populations. Gene positional information was based on the GRCh38 genome annotation. Analysis was performed with default parameters, including denoise = TRUE and HMM = TRUE to enhance CNV signal detection. Cells showing broad chromosomal gains or losses were classified as malignant, while those with flat CNV profiles similar to reference cells were considered non-malignant.

CytoTRACE analysis

CytoTRACE was applied to estimate the differentiation potential of tumor cells based on transcriptional diversity. Raw (unnormalized) gene count matrices were used as input, and standard filtering was performed to remove low-quality cells and lowly expressed genes. The analysis was run with default parameters. Each cell was assigned a CytoTRACE score, where higher scores indicate a more primitive, less differentiated state. These scores were projected onto UMAP to visualize developmental hierarchies within the tumor cell compartment. Cells with the highest CytoTRACE scores were interpreted as possessing higher plasticity and potential malignancy.

Gene-set enrichment analysis (GSEA)

GSEA was used to analysis the function of RILPL2. TCGA-CESC cohort was divided into two groups based on median value of RILPL2 expression. We ranked DEGs between two groups by their association with RILPL2 ^high^ expression group and RILPL2 ^low^ expression group. RILPL2 ^high^ expression group was enriched in the expression signature of GO-BP, KEGG, and Hallmark.

**Plasmids, shRNA, siRNA and transfection**

Flag-tagged RILPL2, HA-tagged LDHA, Myc-tagged TRIM21, and His-tagged ubiquitin were generated by cloning the corresponding coding sequences into the pCDH-CMV-MCS-EF1α-Puro vector with an N-terminal Flag, HA, Myc, or His tag, respectively. C28A mutant plasmid of TRIM21 was constructed in house by using QuickMutation™ Site-Directed Mutagenesis Kit (Beyotime Biotechnology, Shanghai, China). The shRNAs targeting RILPL2 and siRNA targeting LDHA and TRIM21 were obtained from GenePharma (Shanghai, China). Cells were transfected with the indicated plasmids, shRNAs, and siRNAs with Lipofectamine 3000 (Invitrogen) according to the manufacturer’s instructions. RT-qPCR and Western blot were used to detect overexpression and silencing efficiency of lentivirus-mediated RILPL2 in indicated cells. Knockdown sequences for the target genes are provided in Supplementary Table S9.

**Quantitative real-time PCR (qRT-PCR)**

Total RNA was extracted using RNA-easy Isolation Reagent (Vazyme Biotech, China). RNA concentration and purity were determined with a NanoDrop 2000 spectrophotometer, and samples with A260/A280 ratios of 1.8–2.0 were used. Subsequently, 1 µg of RNA was reverse transcribed into cDNA using the PrimeScript RT Reagent Kit with gDNA Eraser (Takara). Quantitative real-time PCR was performed with TB Green® Premix Ex Taq II (Takara) on a QuantStudio™ 7 Flex Real-Time PCR system. Relative gene expression was calculated using the 2^−ΔΔCt method, with β-actin as the internal control. Primer sequences for the target and reference genes are provided in Supplementary Table S10.

**Western blotting**

Cells were lysed on ice using RIPA buffer (Beyotime Biotechnology, China), and protein concentrations were determined by a BCA assay (Pierce, USA). Equal amounts of protein (50 μg) were separated by 10% SDS-PAGE and transferred onto PVDF membranes (Millipore, USA). Membranes were blocked with 5% skim milk at room temperature and then incubated with primary antibodies at 4 °C overnight. After washing with PBST, membranes were incubated with HRP-conjugated secondary antibodies at room temperature. Protein signals were visualized using an ECL detection system. Antibodies used in this study are listed in Supplementary Table S11.

**Immunofluorescence**

Cells were inoculated onto Cover Glasses (NEST Biotechnology Co. Ltd, China), left to stretched, fixed in 4% paraformaldehyde for 20 min, penetrated with 0.25% Triton X-100, and blocked in 5% bovine serum albumin for 1 h. Then, cells were incubated with primary antibodies at 4°C overnight and exposed to fluorochrome-labeled second antibodies for 1 h at room temperature. After staining with DAPI for 5 min, cells were imaged under confocal laser scanning microscope (PerkinElmer, USA). The antibodies used are listed in Supplementary Table S11.

**CHX chase assay**

The effect of RILPL2 on the stability of LDHA was tested using the CHX assay. The indicated cells were treated with the protein synthesis inhibitor CHX (Sigma) for the indicated durations before collection.

**Co-IP Assay**

A Co-IP Kit (Thermo Fisher, USA) was used for the Co-IP assay. Cell lysates were prepared in lysis buffer and incubated with agarose bead-conjugated antibodies overnight at 4 °C with gentle rotation. After washing with lysis buffer, proteins that bound to the beads were eluted with elution buffer for western blotting.

**Molecular docking**

In order to predict the model of the direct binding between the three protein molecules RILPL2, TRIM21 and LDHA, we first obtained the 3D spatial structures of these proteins through SWISS-MODE and PDB databases, and then based on the computational protein docking method, using Cluspro (<https://cluspro.bu.edu/>) online tool predicts the most likely complex model for RILPL2, TRIM21 and LDHA binding. The interaction surfaces in protein complexes were further analyzed online by PDBePISA (<https://www.ebi.ac.uk/msd-srv/prot_int/pistart.html>). Finally, conformational mapping and docking region analysis were performed using PYMOL (<https://pymol.org/>) and LIGPLOT (<https://www.ebi.ac.uk/thornton-srv/software/LigPlus/>).

**Histone extraction and chromatin immunoprecipitation assay (ChIP)**

EpiQuik Total Histone Extraction Kit (Epigentek, USA) was used to isolate histones from cells following the manufacturer’s instructions. For ChIP assay, cells were fixed with 1% formaldehyde for 10 min and the fixation was stopped with 0.125 M glycine. Then, the cell lysis buffer was added, and the samples were sonicated to generate 200 to 1,000 bp DNA fragments. The resulting cell lysates were immunoprecipitated using indicated antibodies and analyzed via ChIP-qPCR. The primers used are shown in Supplementary Table S12.

**Sequence of RNA (RNA-Seq)**

Total RNA was isolated from Hela cells with knockdown of RILPL2 and corresponding control (each in three replicates) for RNA-seq via the Nanjing Jiangbei New Area Biopharmaceutical Public Service Platform (Nanjing, China). A cDNA library was created from RNA samples of high quality. To create the final cDNA library, 12–15 cycles of PCR were applied to the purified products. RNA-Seq was performed on Illumina NovaSeq 6000 libraries according to the manufacturer’s instructions to screen DEGs for a Log2FC > 0.5 and *P* < 0.05.

**Immunohistochemistry (IHC)**

For immunohistochemistry, tissues were heated at 65°C for 3 h, then dewaxed and hydrated, and the samples were subjected to EDTA-mediated high-temperature antigen retrieval. The samples were then incubated overnight at 4°C with primary antibodies. After washing 3 times, the second antibody was then added and incubated at room temperature for 2 h. The staining was scored according to the staining intensity and the distribution of stained cells. The IHC sections were further scanned with NanoZoomer S60 (Hamamatsu Photonics)

**Code availability**

The codes employed for the bioinformatics analyses in this paper are not publicly available due to data security considerations but can be obtained from the corresponding author upon reasonable request.

**Supplementary Figures**

**Fig. S1**


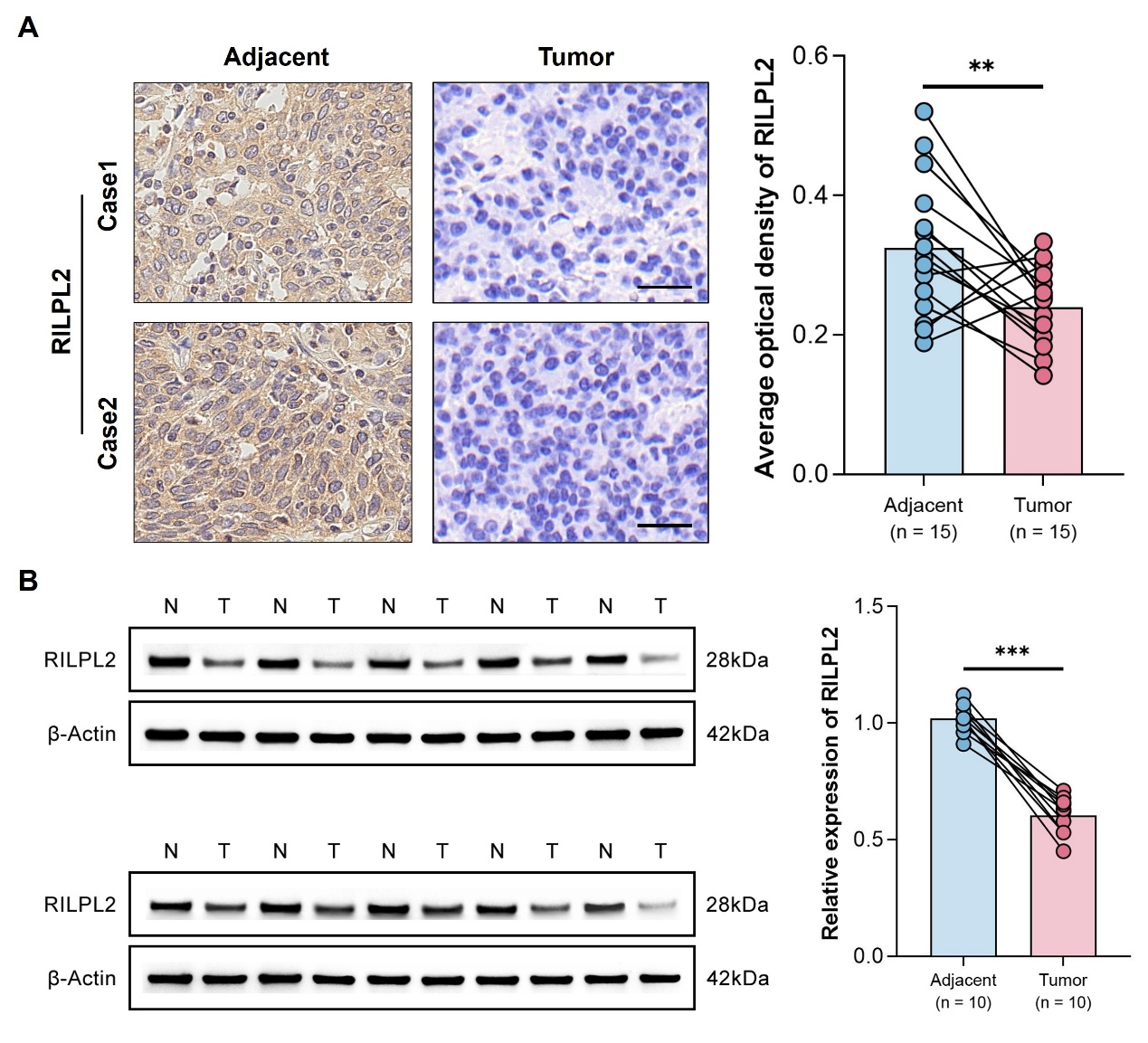


**Fig. S1** RILPL2 is downregulated in cervical cancer (CC).

(A) Representative images and statistical graph (n = 15, **P<0.01, paired two-tailed Student’s t-test) of IHC staining analysis of RILPL2 protein levels in CC tissues and paired adjacent tissues. Scale bar, 100 μm. (B) The protein level of RILPL2 and quantification graph (n = 10, ***P<0.001, paired two-tailed Student’s t-test) was detected in clinical specimens by Western blot.

**Fig. S2**


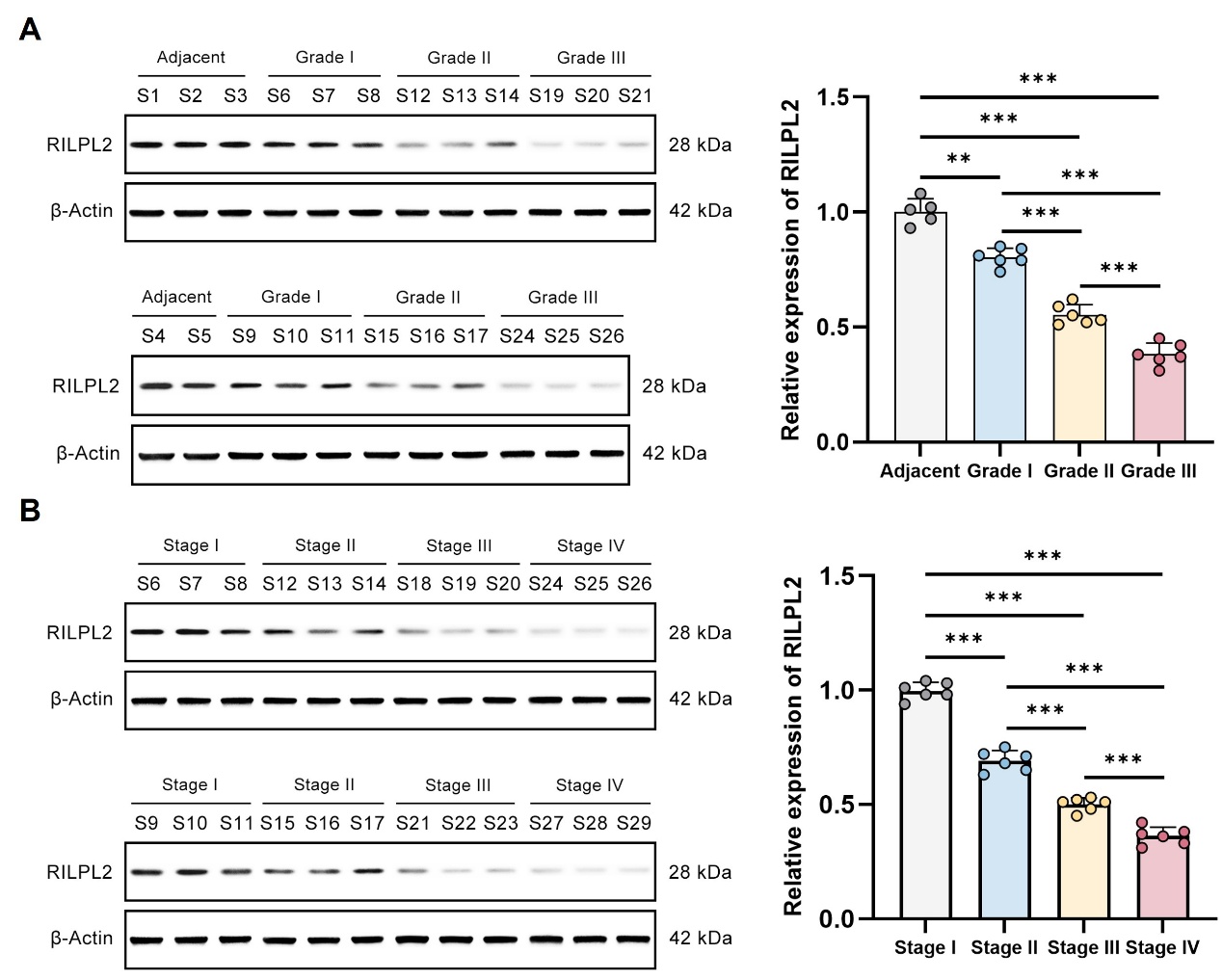


**Fig. S2 RILPL2 protein level was negatively correlated with clinical characteristics of CC.**

(A) The protein level of RILPL2 and quantification graph (**P<0.01, ***P<0.001, one-way ANOVA with post hoc test) was detected in clinical specimens with different pathological grades (Adjacent, Grades I, II, and III) by Western blot. (B) The protein level of RILPL2 and quantification graph (***P<0.001, one-way ANOVA with post hoc test) was detected in clinical specimens at different stages (7th edition of the AJCC: Stage I, II, III, and IV) by Western blot.

**Fig. S3**


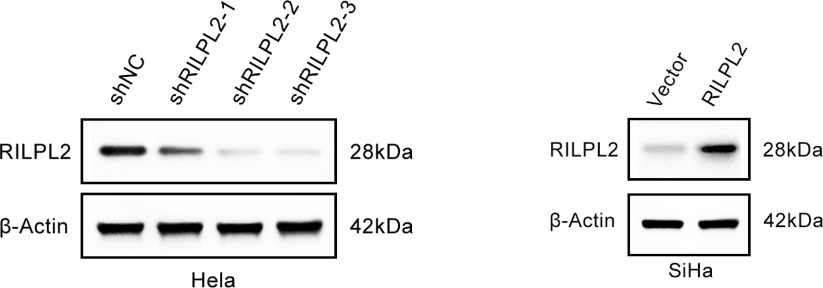


**Fig. S3 Transfection efficiency was examined by western blot analysis.**

**Fig. S4**


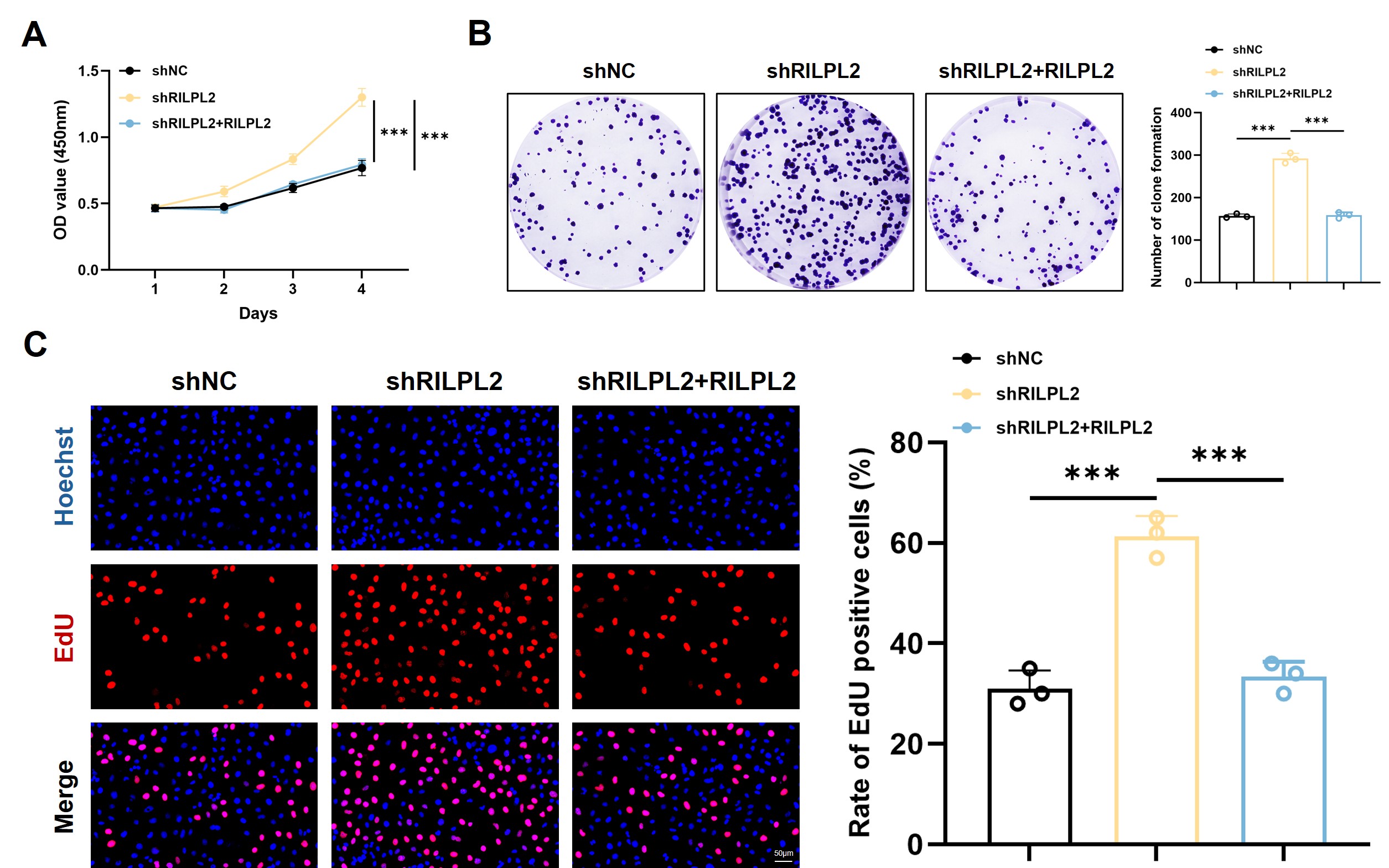


**Fig. S4 Rescue experiments on cell proliferation were performed by re-expressing RILPL2 in RILPL2-silenced Hela cells.**

The effect of RILPL2 on proliferation in RILPL2-silenced Hela cells using (A) CCK-8 (n = 3 independent experiments, ***P<0.001, two-way ANOVA with post hoc test), (B) colony formation, and (C) EdU assay (n = 3 independent experiments, ***P<0.001, one-way ANOVA with post hoc test). Scale bar, 50 μm.

**Fig. S5**


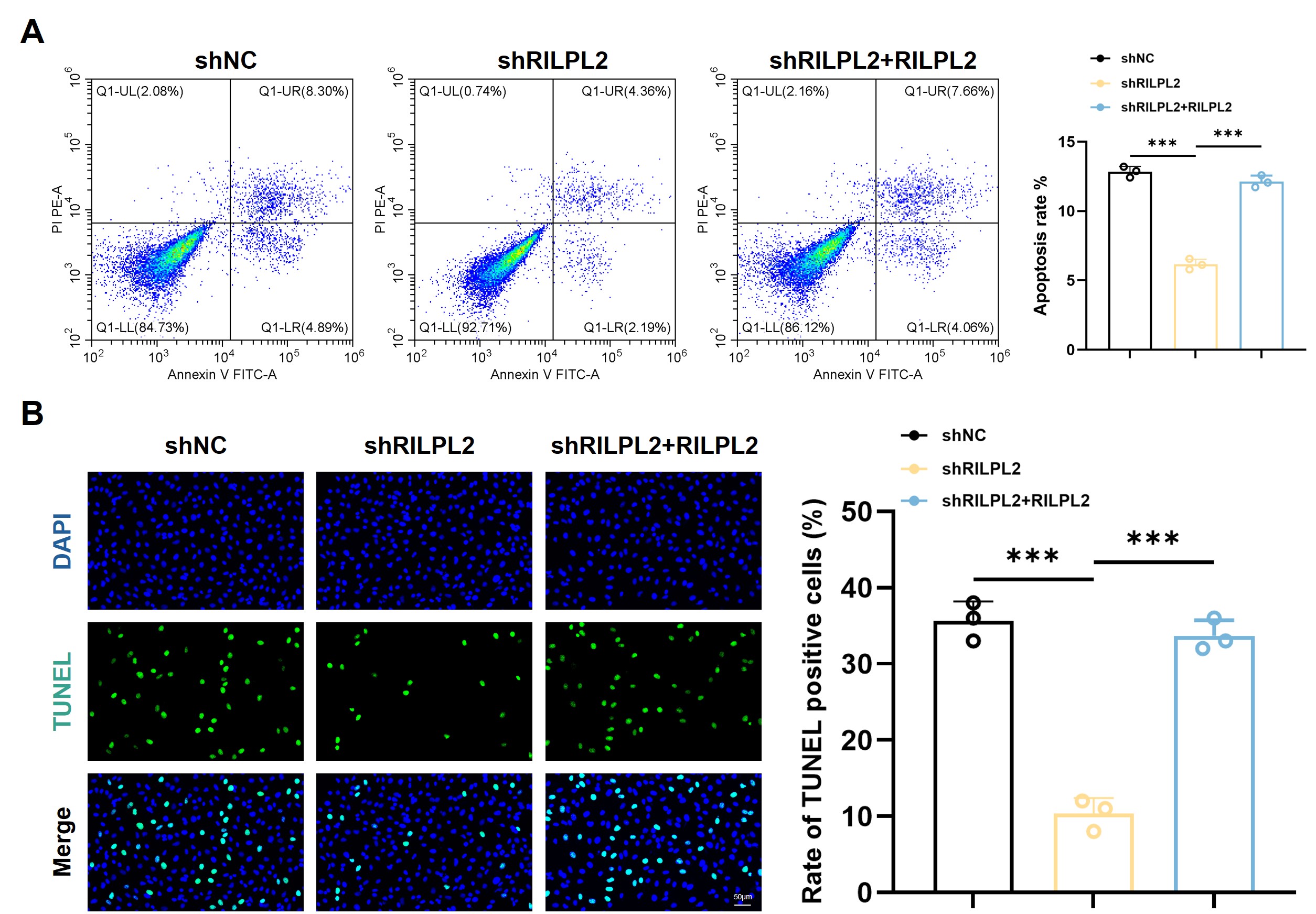


**Fig. S5 Rescue experiments on cell apoptosis were performed by re-expressing RILPL2 in RILPL2-silenced Hela cells.**

The effect of RILPL2 on cell apoptosis was evaluated by (A) flow cytometric analysis and (B) TUNEL assay (n = 3 independent experiments, ***P<0.001, one-way ANOVA with post hoc test). Scale bar, 50 μm.

**Fig. S6**


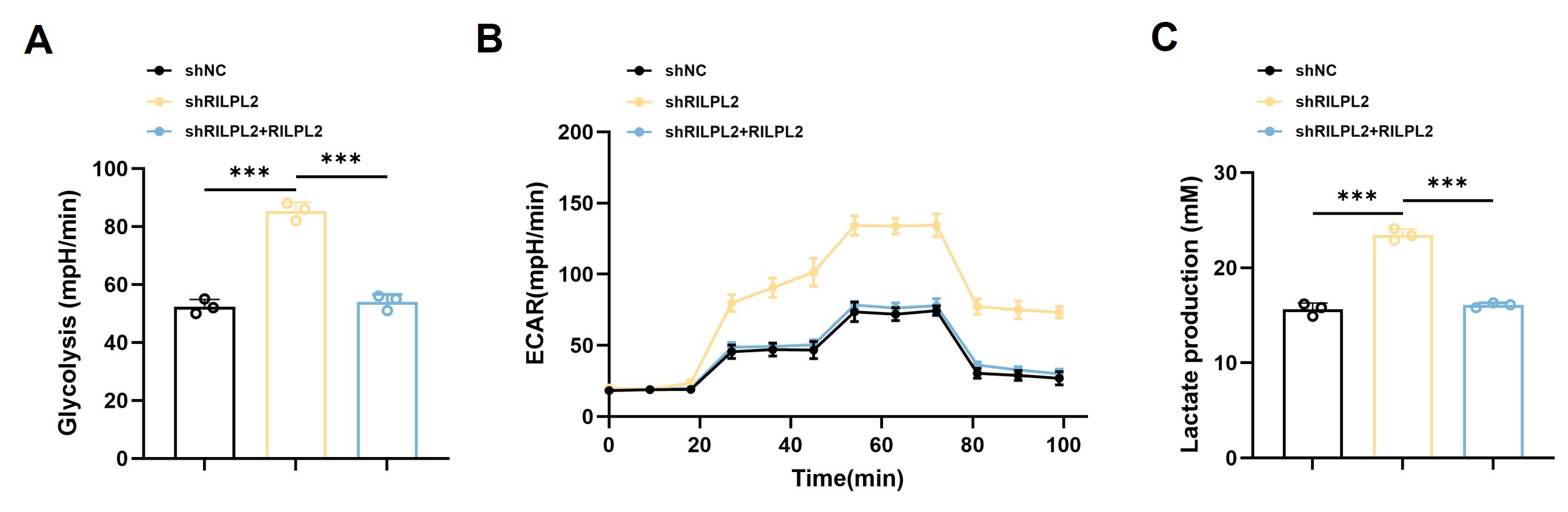


**Fig. S6 Rescue experiments on glycolysis were performed by re-expressing RILPL2 in RILPL2-silenced Hela cells.**

The effect of RILPL2 on glycolysis in vitro was measured by (A) glycolytic activity assay (n = 3 independent experiments, ***P<0.001, one-way ANOVA with post hoc test), (B) extracellular acidification rate (ECAR) analysis, and (C) lactate production assay (n = 3, ***P<0.001, one-way ANOVA with post hoc test).

**Fig. S7**


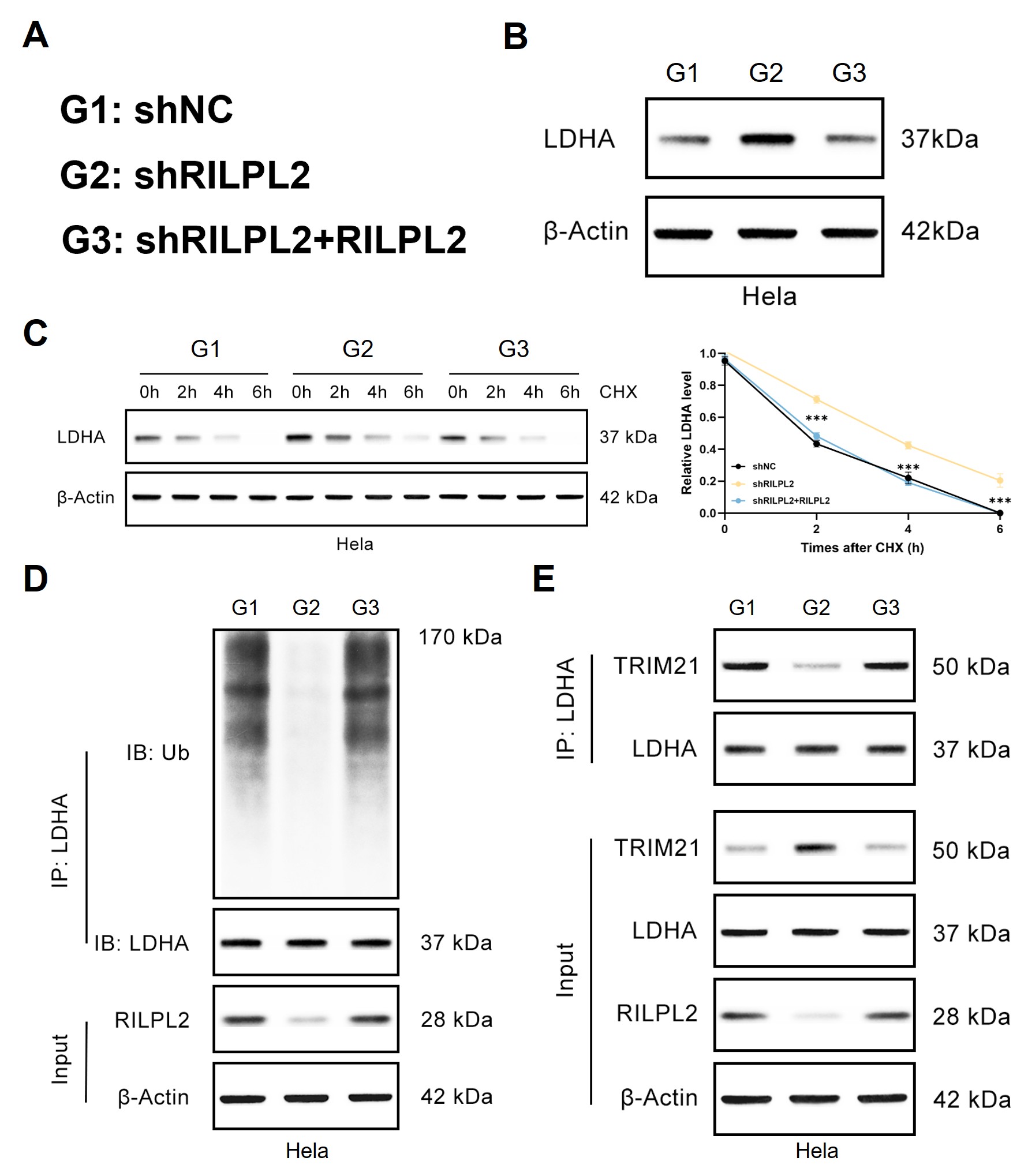


**Fig. S7 Rescue experiments on Western blot were performed by re-expressing RILPL2 in RILPL2-silenced Hela cells.**

(A) Rescue experiments groups design. (B) Detecting LDHA protein levels in Hela cells in indicated groups by Western blot. (C) Representative images and the corresponding quantification of the Western blot analysis (n = 3 independent experiments, ***P<0.001, two-way ANOVA with post hoc test) were conducted to assess the half-life of the LDHA protein in Hela cells. (D) Assessment of LDHA ubiquitination in indicated groups. (E) Assessment of Co-IP of LDHA and TRIM21 in indicated groups.

**Fig. S8**


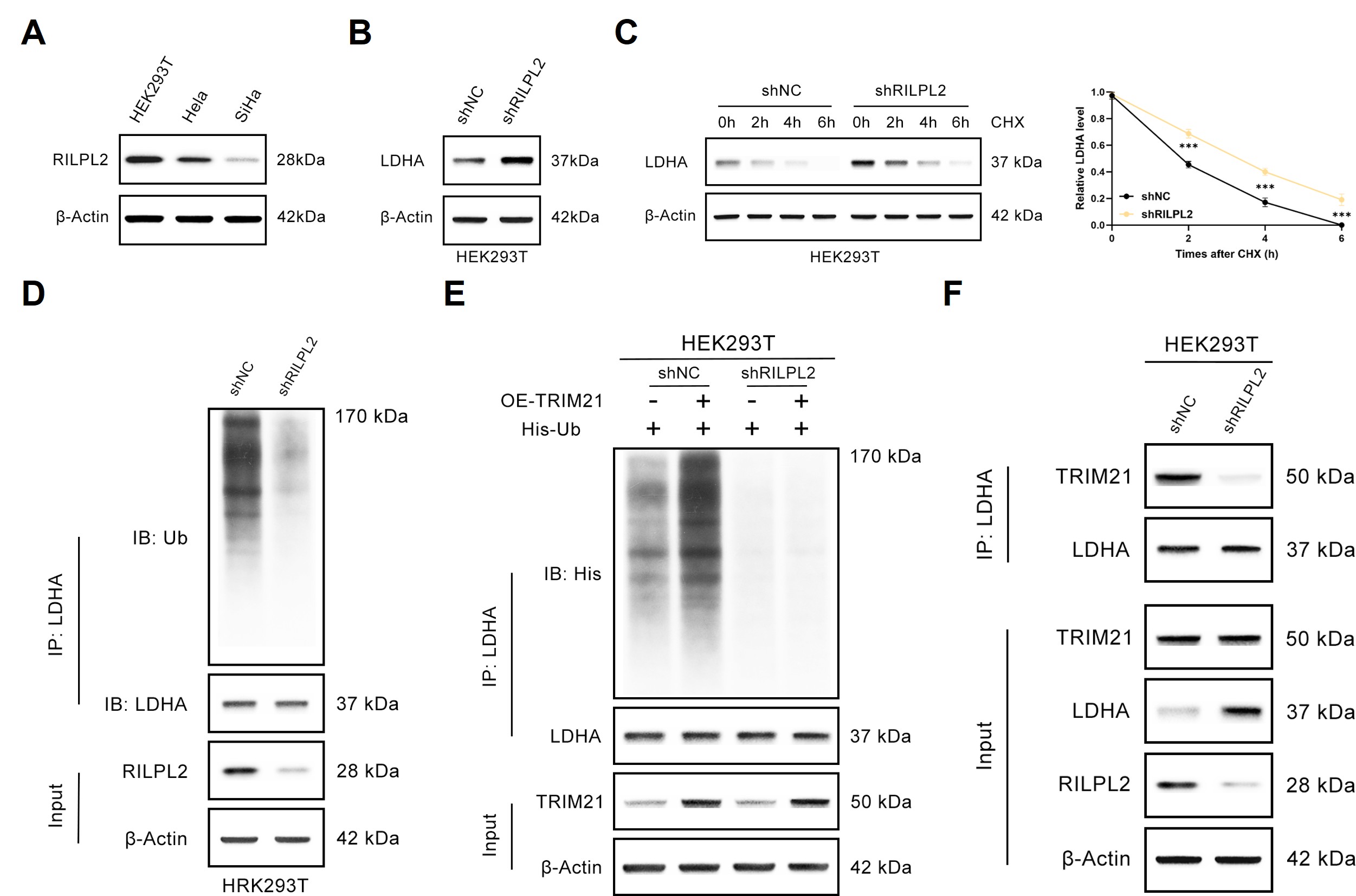


**Fig. S8 RILPL2 facilitates LDHA degradation through TRIM21-mediated ubiquitination.**

(A) Detecting RILPL2 protein levels in HEK293T cells and CC cell lines (Hela and SiHa) by Western blot. (B) Western blot analysis of LDHA expression with RILPL2 knockdown in HEK293T cells. (C) Representative images and the corresponding quantification of the Western blot analysis (n = 3 independent experiments, ***P<0.001, two-way ANOVA with post hoc test) were conducted to assess the half-life of the LDHA protein in HEK293T cells. (D) Assessment of LDHA ubiquitination with RILPL2 knockdown in HEK293T cells. (E) Ubiquitination level of LDHA in HEK293T-shNC/ HEK293T-shRILPL2 cells with TRIM21 overexpression. (F) Assessment of Co-IP of LDHA and TRIM21 in shNC or shRILPL2 in HEK293T cells.

**Fig. S9**


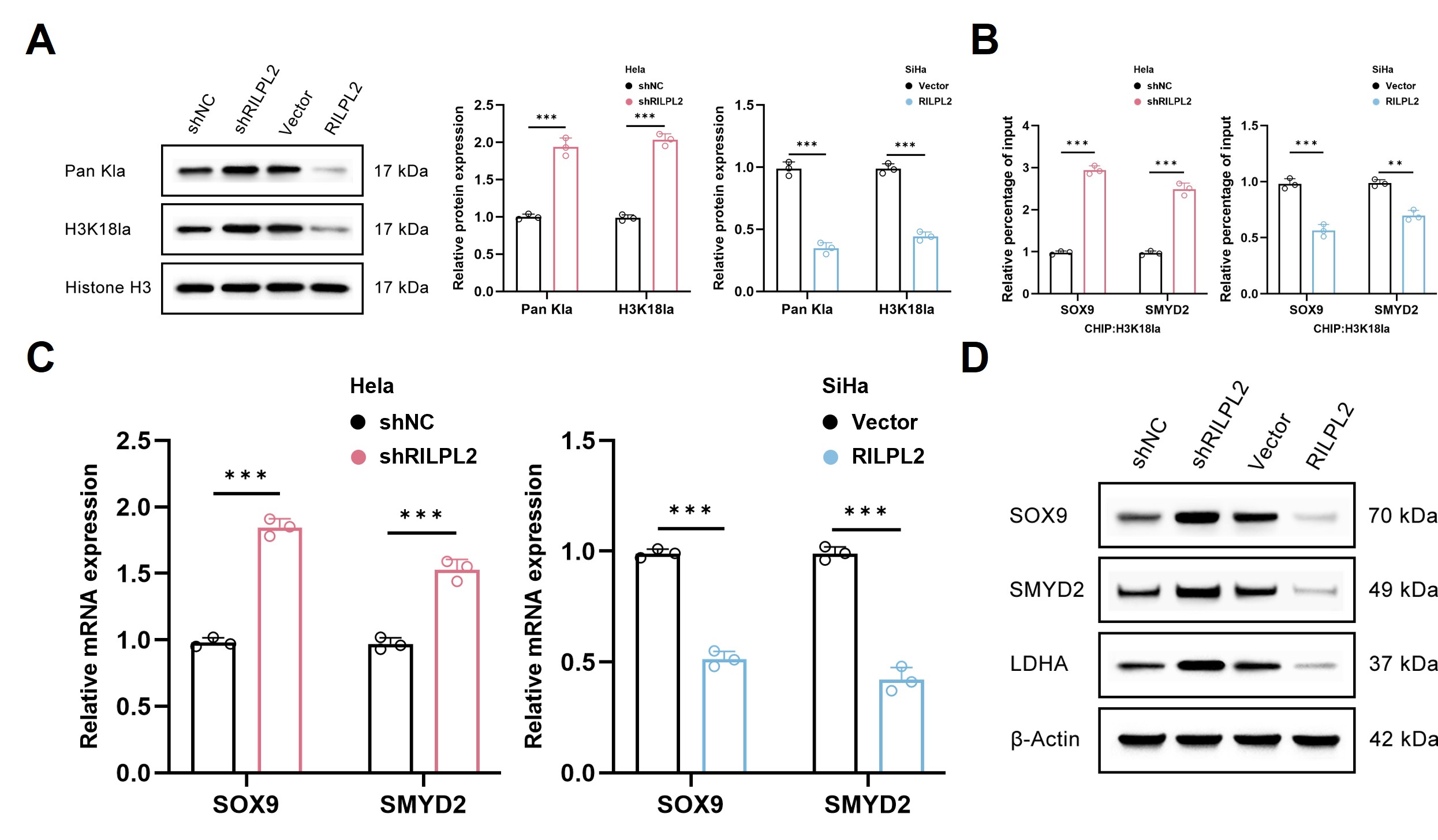


**Fig. S9 RILPL2 blocks H3K18 lactylation in CC cells.**

(A) The levels of pan histone and H3K18 site lactylation were detected by western blot analysis (n = 3 independent experiments, ***P<0.001, unpaired two-tailed Student’s t-test) in different experimental groups. (B) The H3K18la enrichment in SOX9 and SMYD2 was measured by ChIP-qPCR assay (n = 3 independent experiments, **P<0.01, ***P<0.001, unpaired two-tailed Student’s t-test) in different experimental groups. The expression level of SOX9 and SMYD2 was examined by (C) qRT-PCR (n = 3 independent experiments, ***P<0.001, unpaired two-tailed Student’s t-test) and (D) Western blot in different experimental groups.
