# Supplementary material for: RILPL2 suppresses metabolic reprogramming and progression of cervical cancer by attenuating LDHA protein stability and inhibiting H3K18 lactylation
Source: Cell Death Dis. 2026 May 4;17(1):590. doi: 10.1038/s41419-026-08808-9 (PMC13284358; doi:10.1038/s41419-026-08808-9)

Fig. 2B

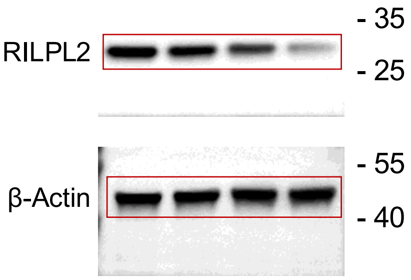

Fig. 4B

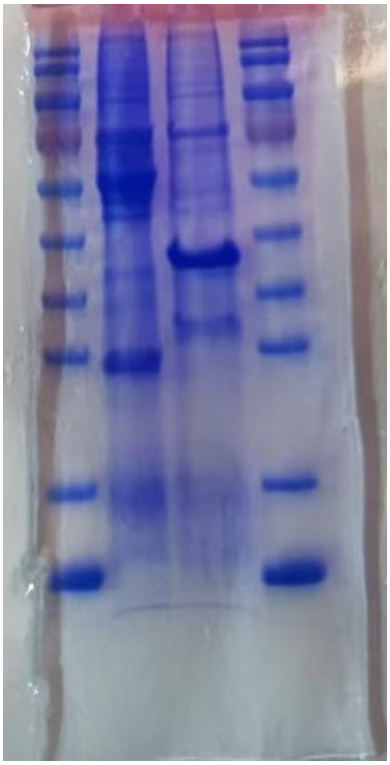

Fig. 4E

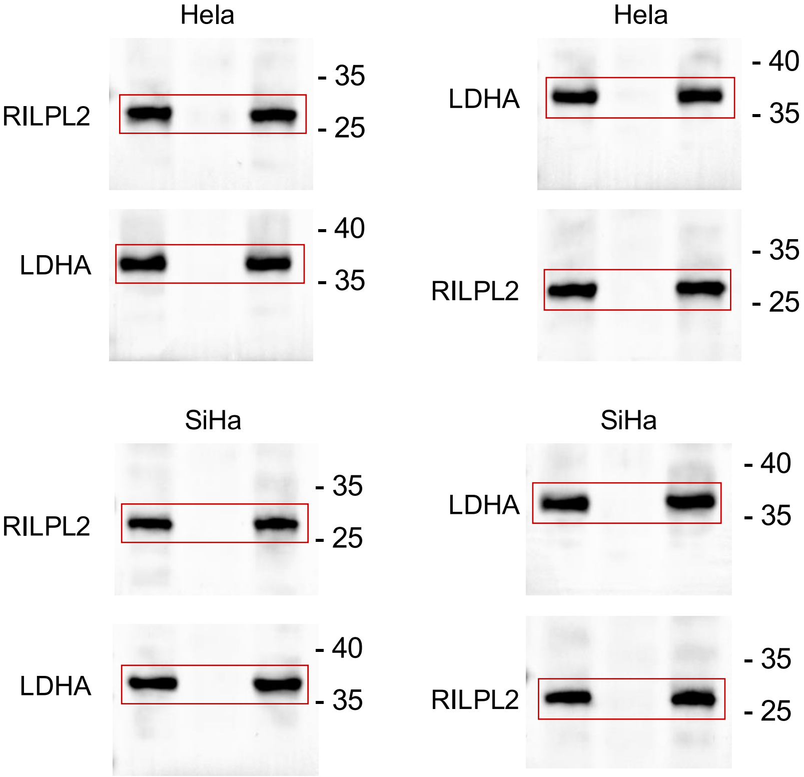

Fig. 6B

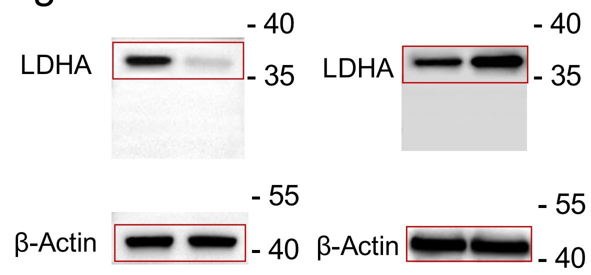

Fig. 6C

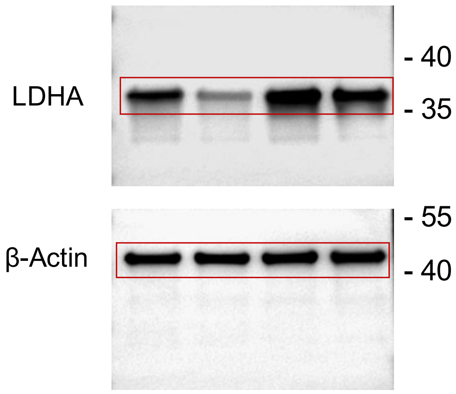

Fig. 6D

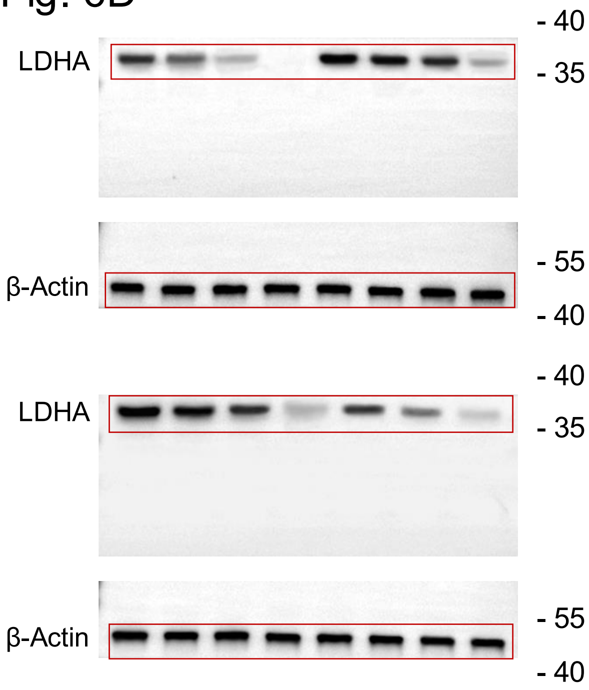

Fig. 6E

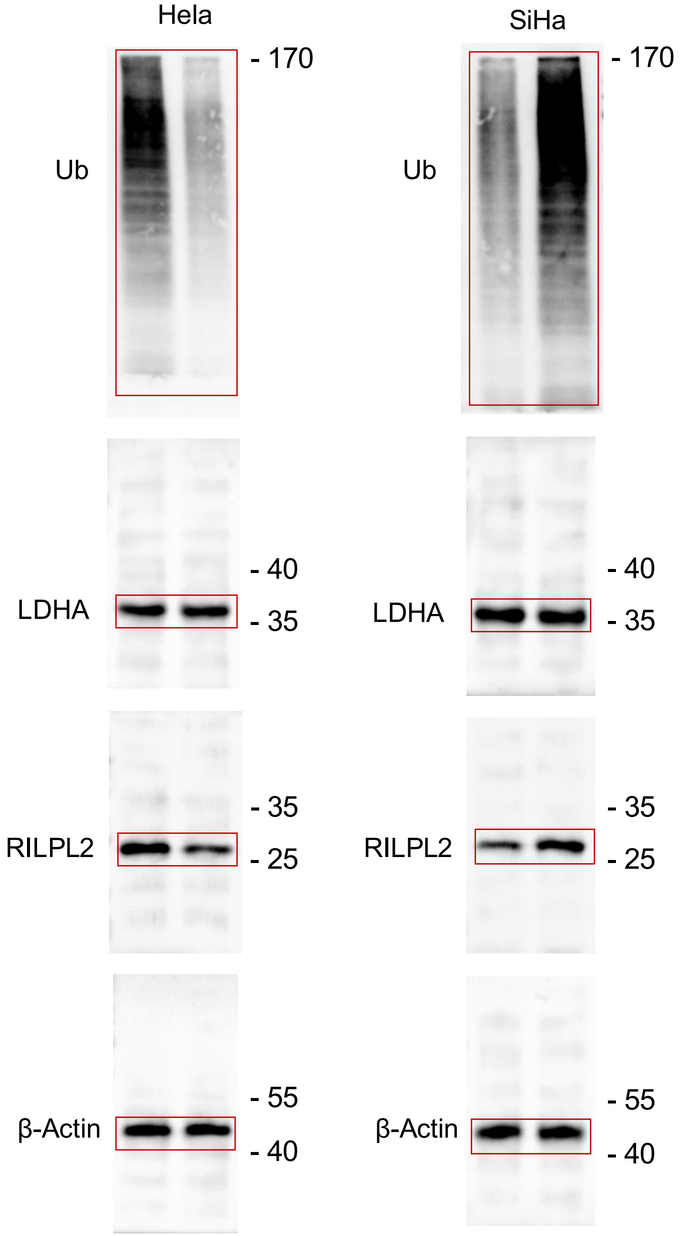

Fig. 6G

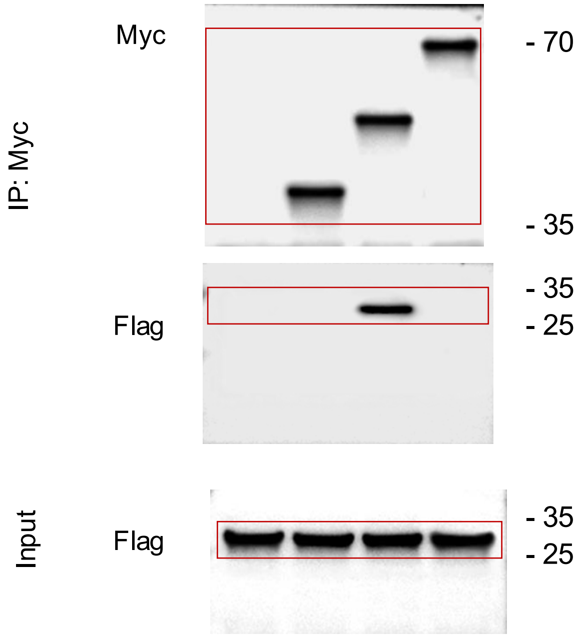

Fig. 6I

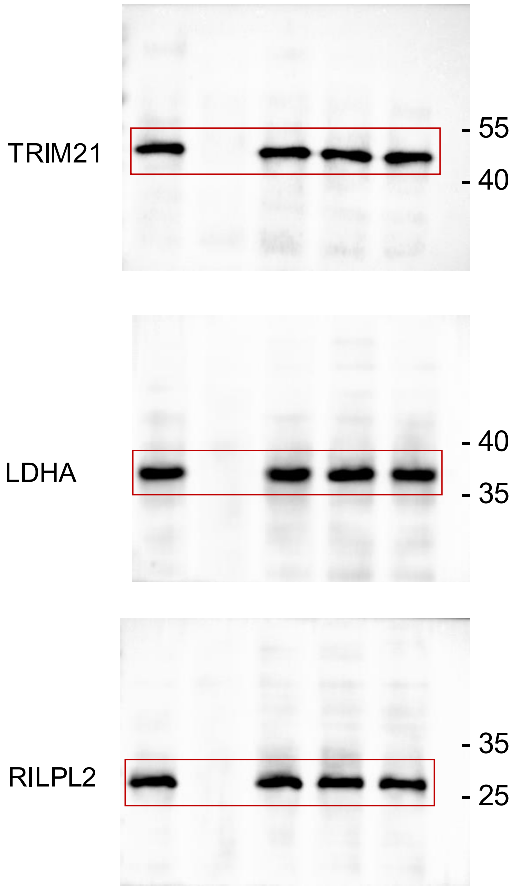
TRIM21
55
40
TRIM21
55
40
LDHA
40
35
LDHA
40
35
RILPL2
35
25
RILPL2
35
25

Fig. 6J

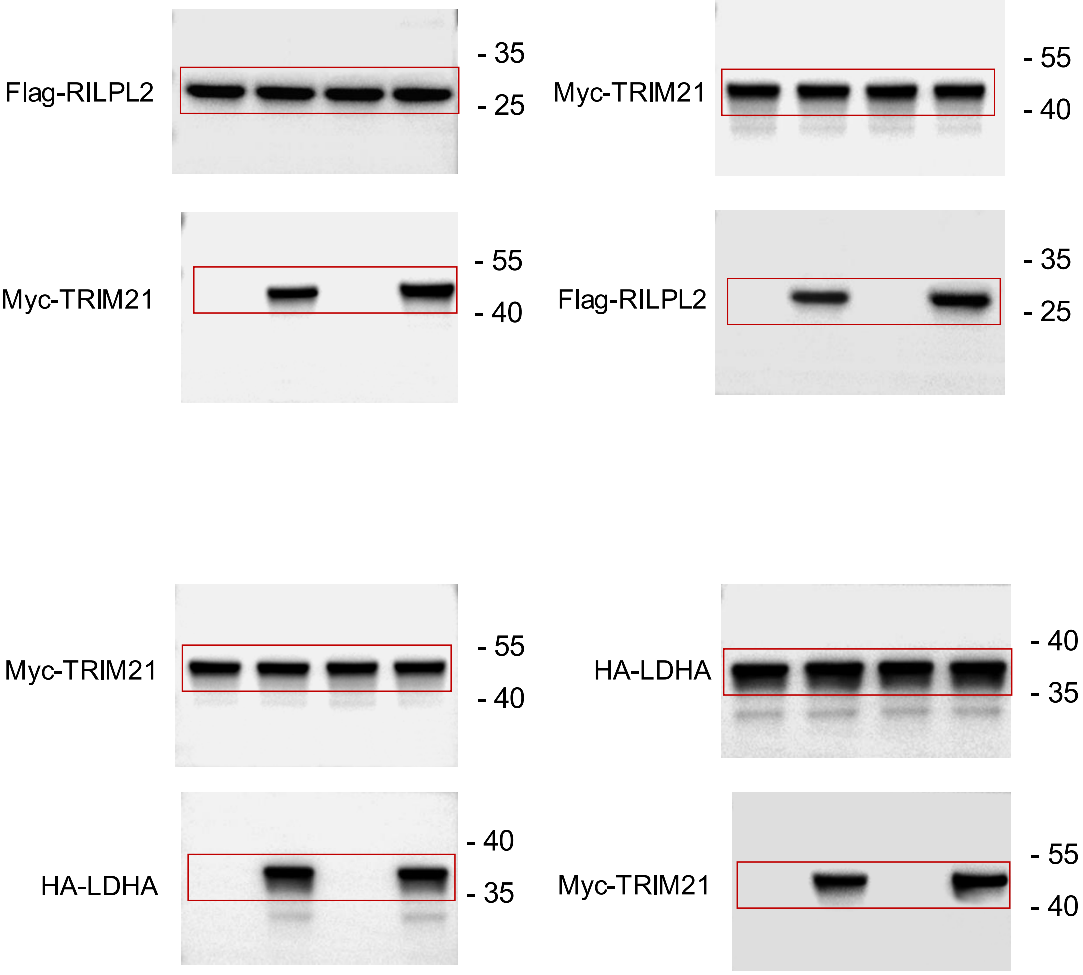
Flag-RILPL2
35
25
Myc-TRIM21
55
40
Myc-TRIM21
55
40
Flag-RILPL2
35
25
Myc-TRIM21
55
40
HA-LDHA
40
35
HA-LDHA
40
35
Myc-TRIM21
55
40

Fig. 6K

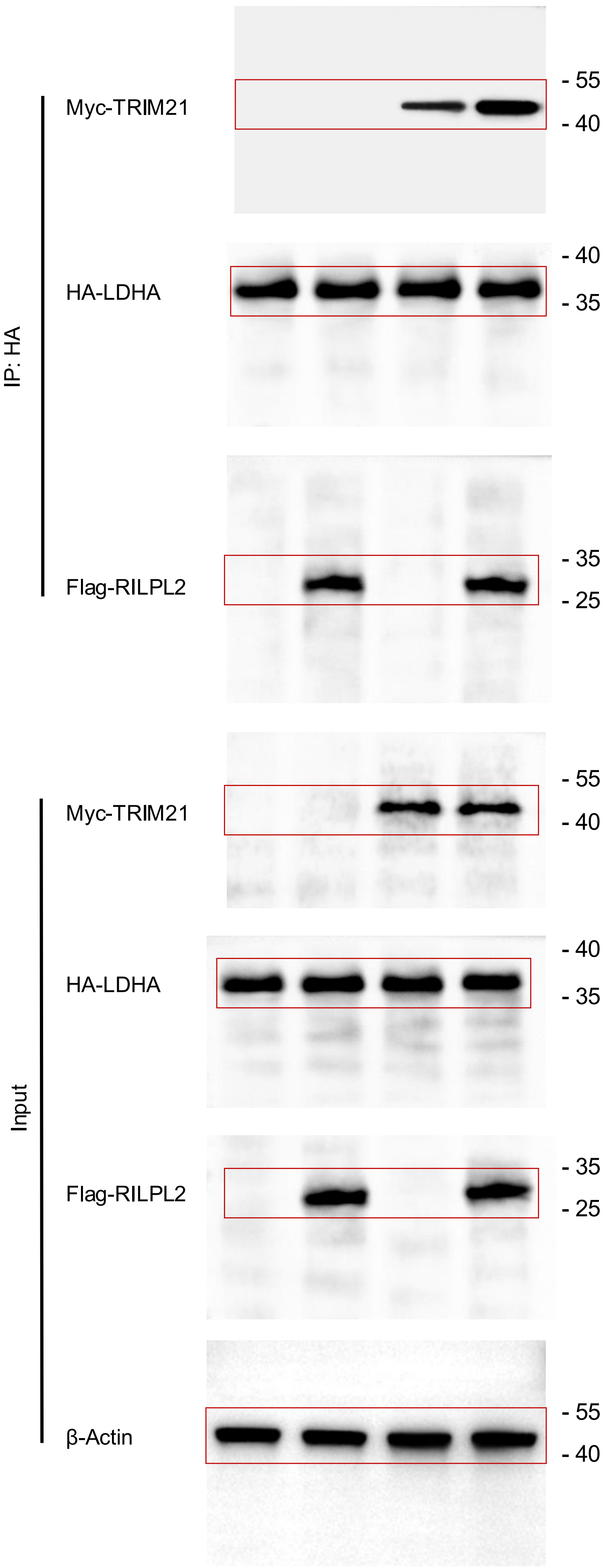
IP: HA
Myc-TRIM21
- 55
- 40
HA-LDHA
- 40
- 35
Flag-RILPL2
- 35
- 25
Input
Myc-TRIM21
- 55
- 40
HA-LDHA
- 40
- 35
Flag-RILPL2
- 35
- 25
β-Actin
- 55
- 40

Fig. 6L

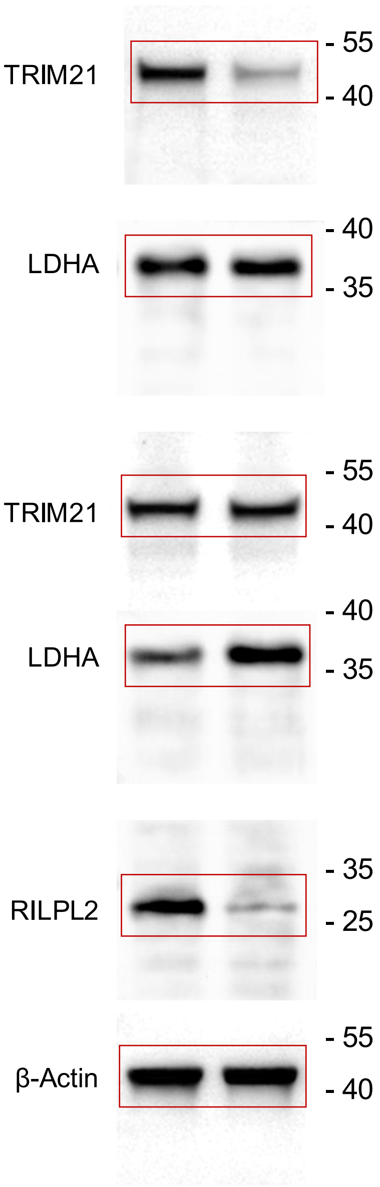
TRIM21
- 55
- 40
LDHA
- 40
- 35
TRIM21
- 55
- 40
LDHA
- 40
- 35
RILPL2
- 35
- 25
β-Actin
- 55
- 40

Fig. 6N

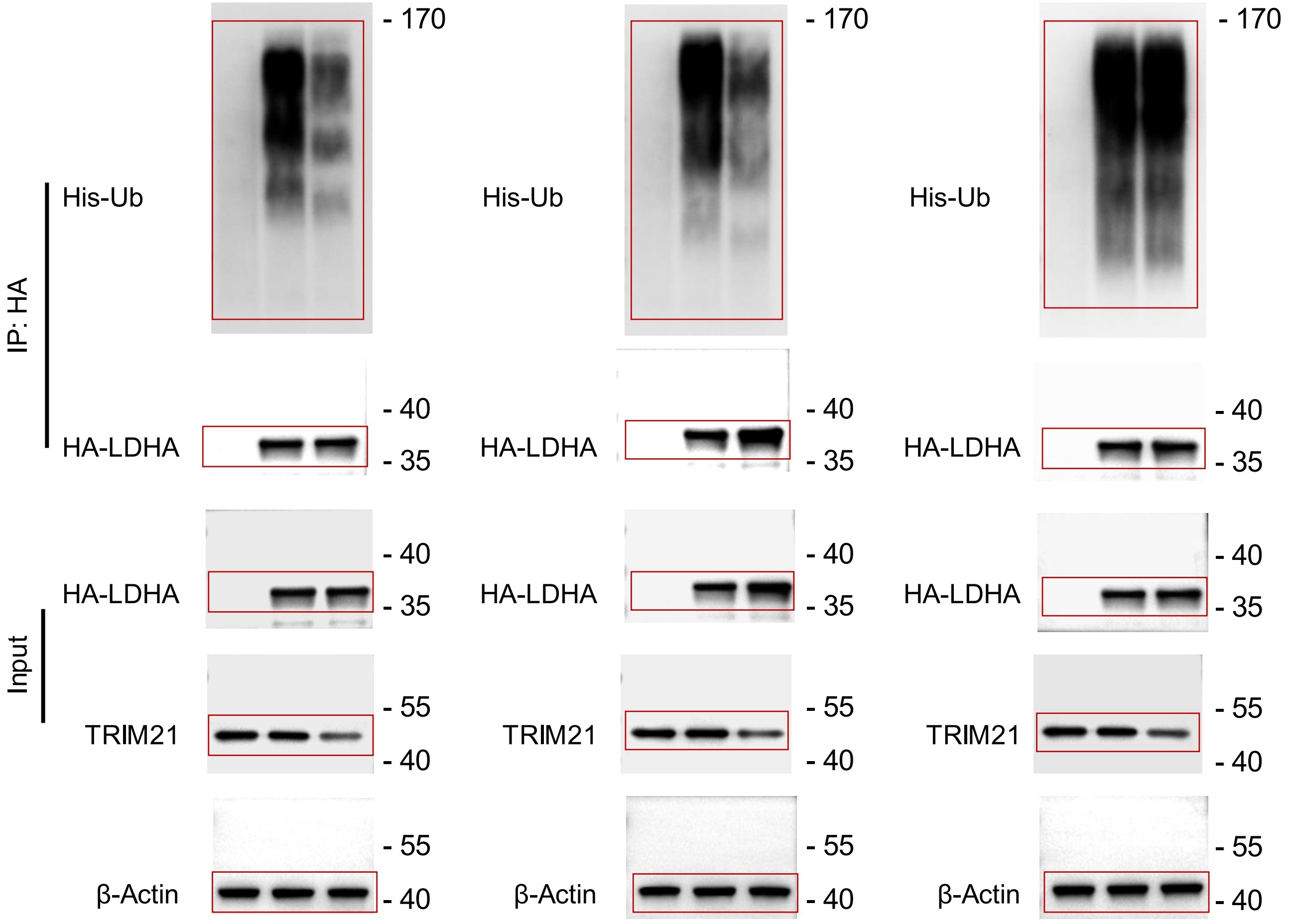

Fig. 6M

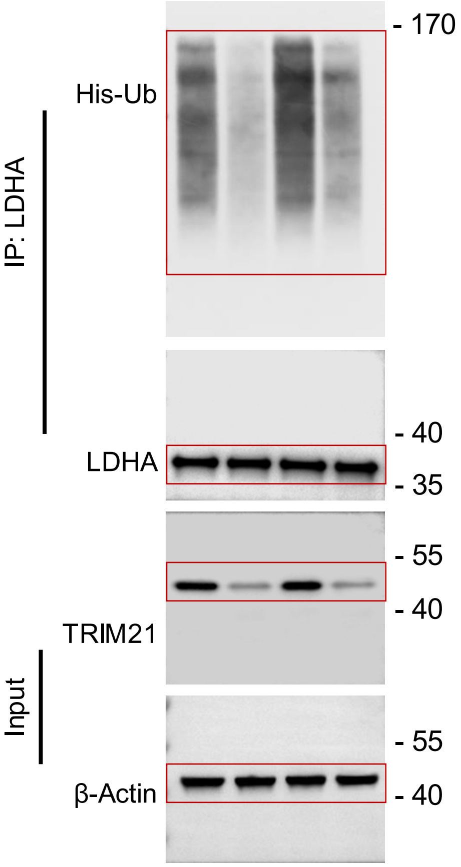

Fig. 6O

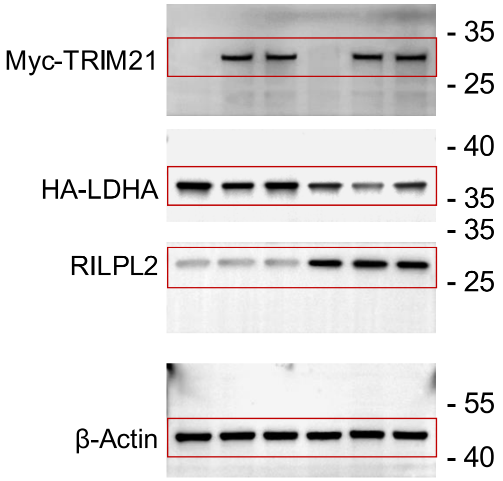

Fig. 7A

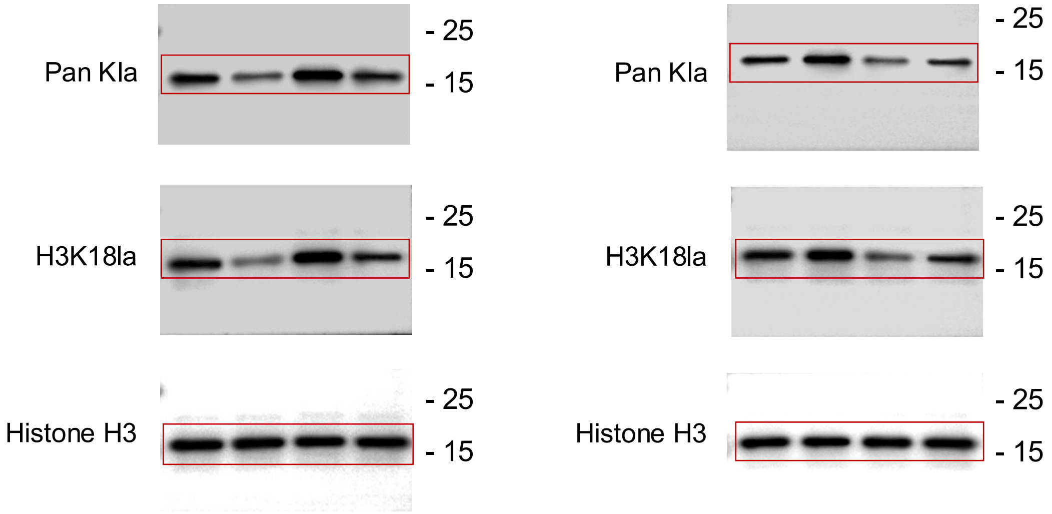

Pan Kla
H3K18la
Histone H3
- 25
- 15

Fig. 7E

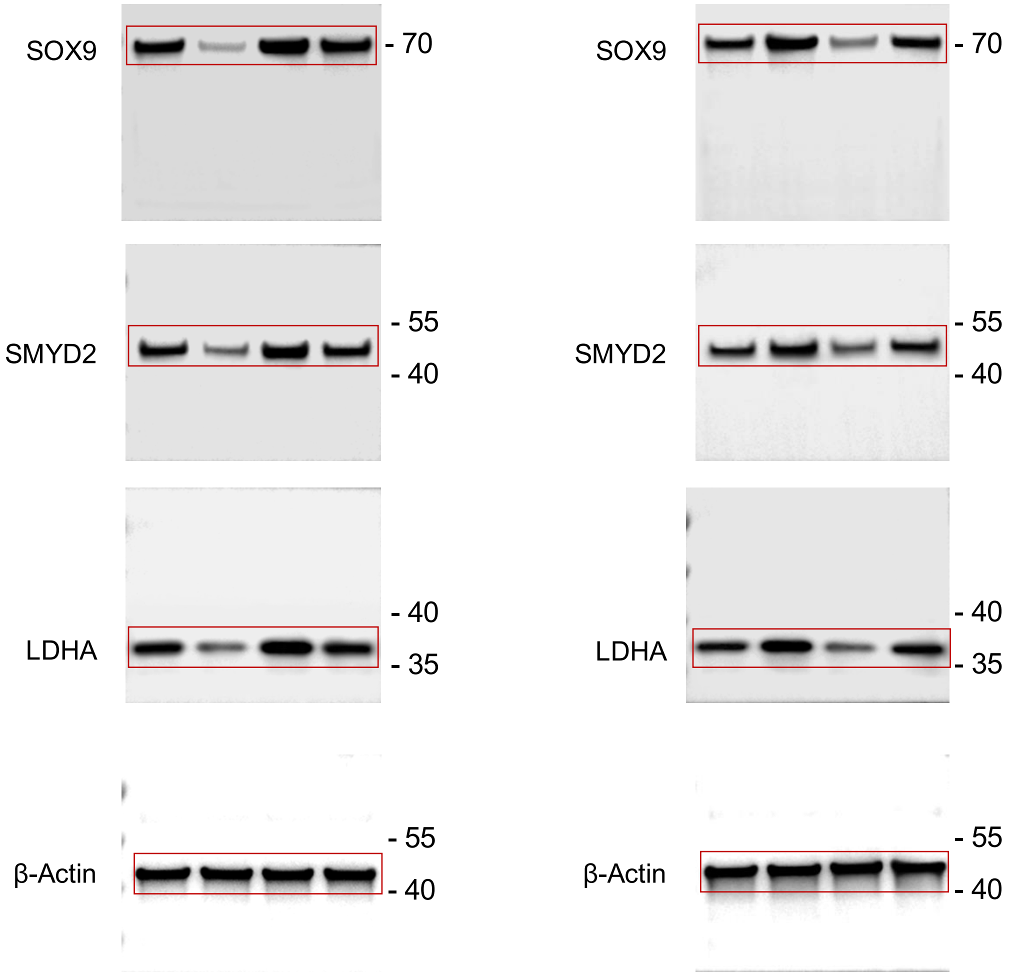

SOX9
SMYD2
LDHA
β-Actin
- 70
- 55
- 40
- 35

Fig. S1B

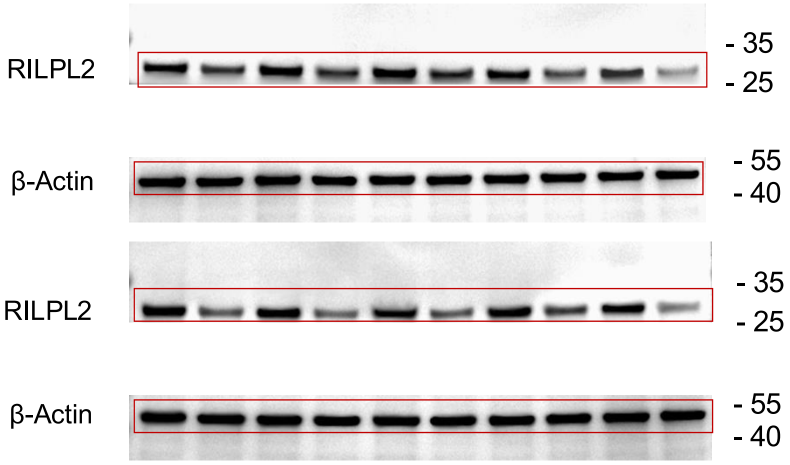

Fig. S2A

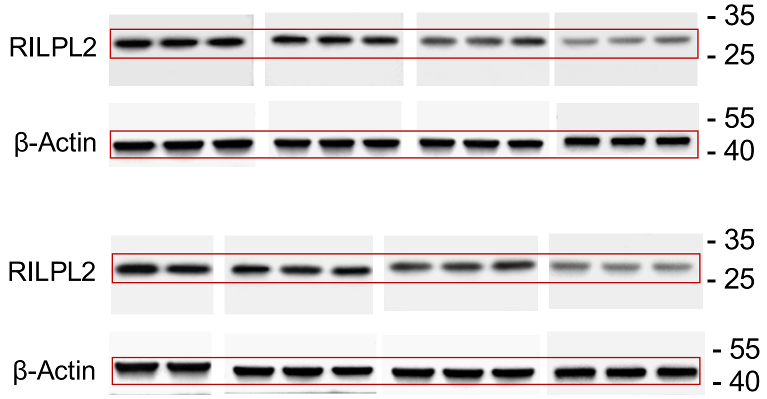

Fig. S2B

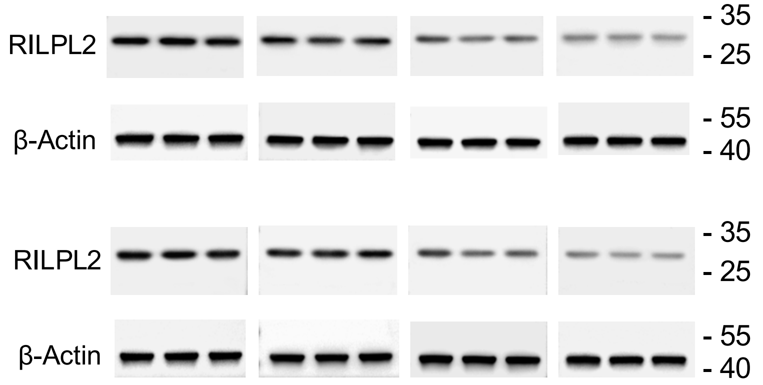

Fig. S3

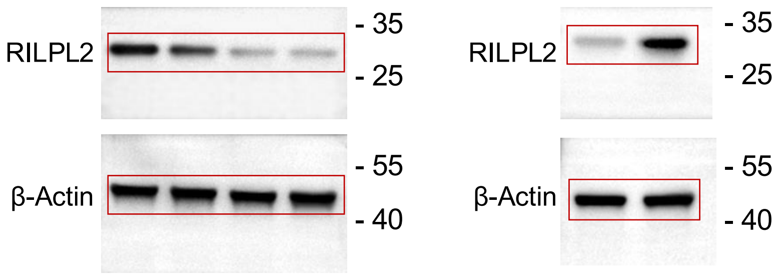

Fig. S7B

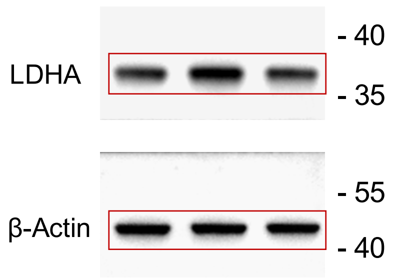

Fig. S7C

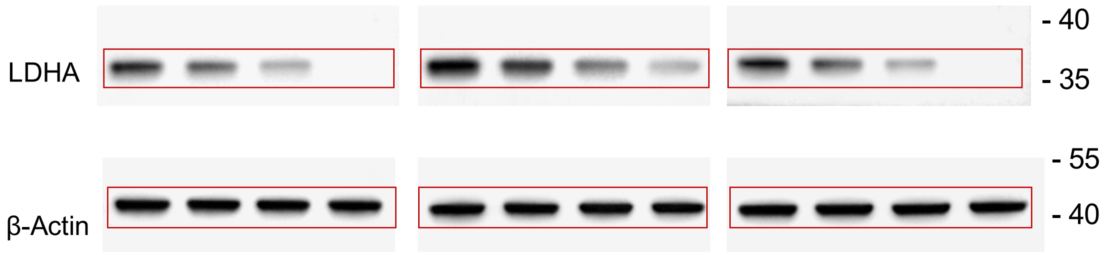

Fig. S7D

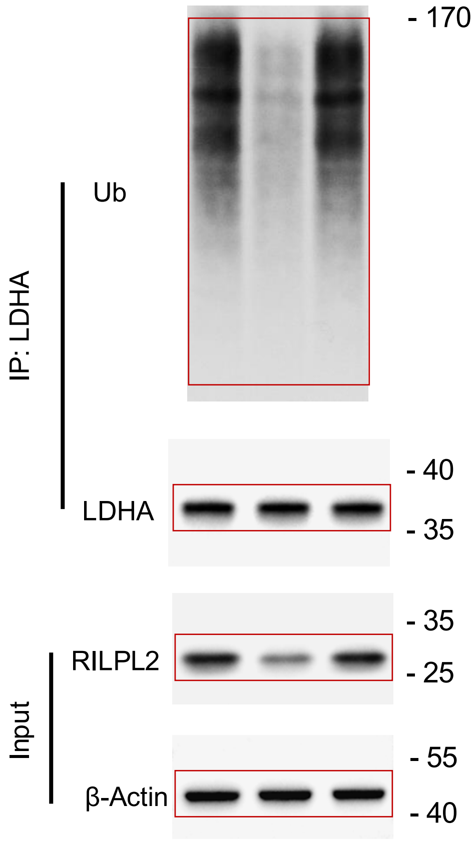

Fig. S7E

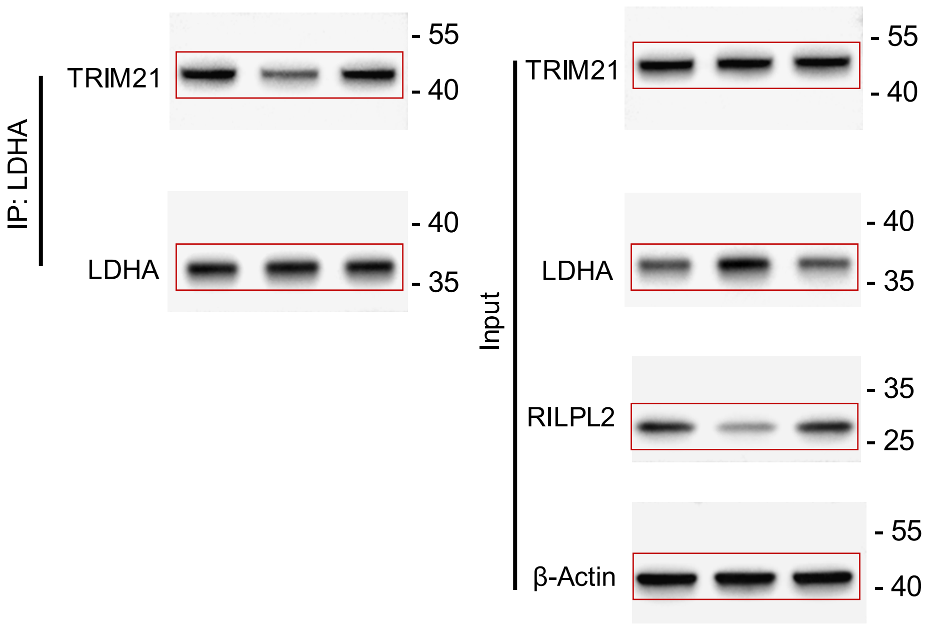

Fig. S8A

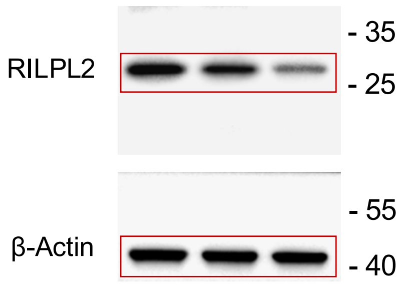

Fig. S8B

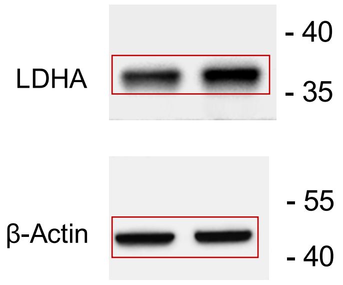

Fig. S8C

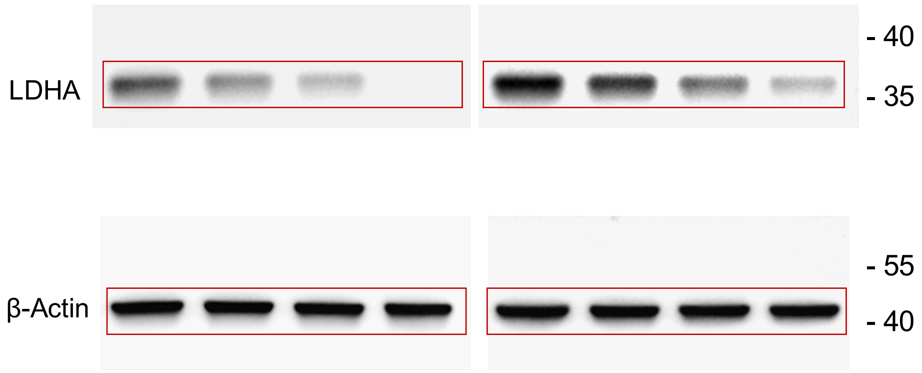

Fig. S8D

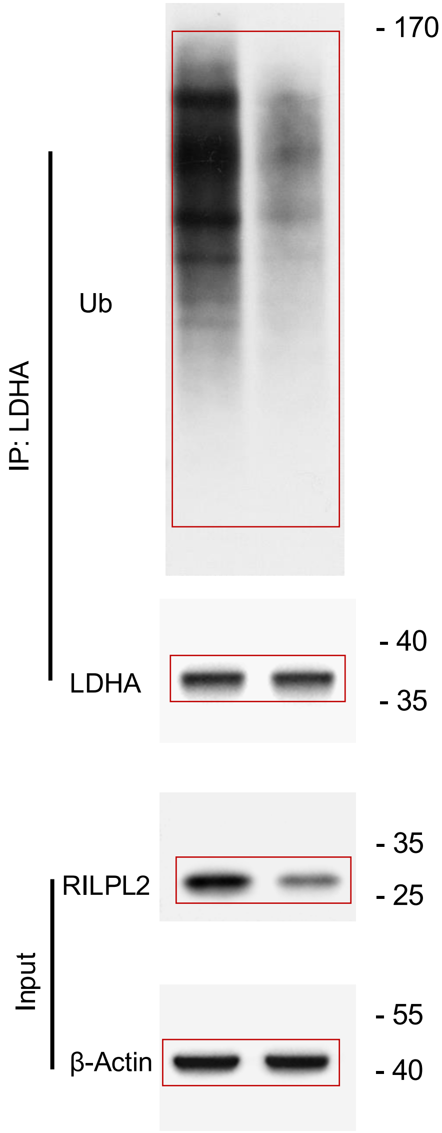

Fig. S8E

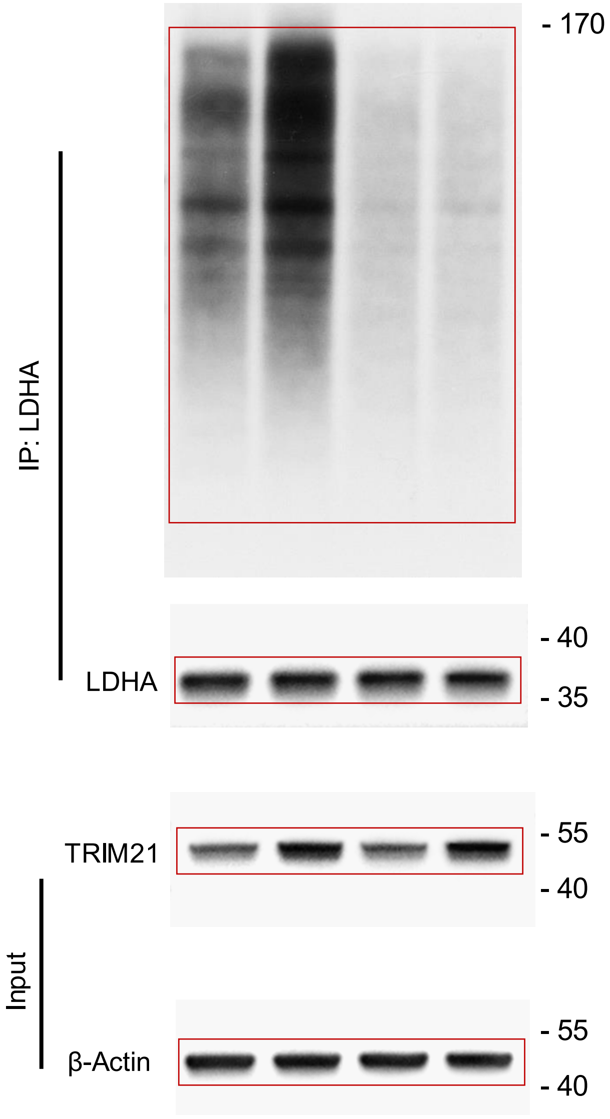

Fig. S8F

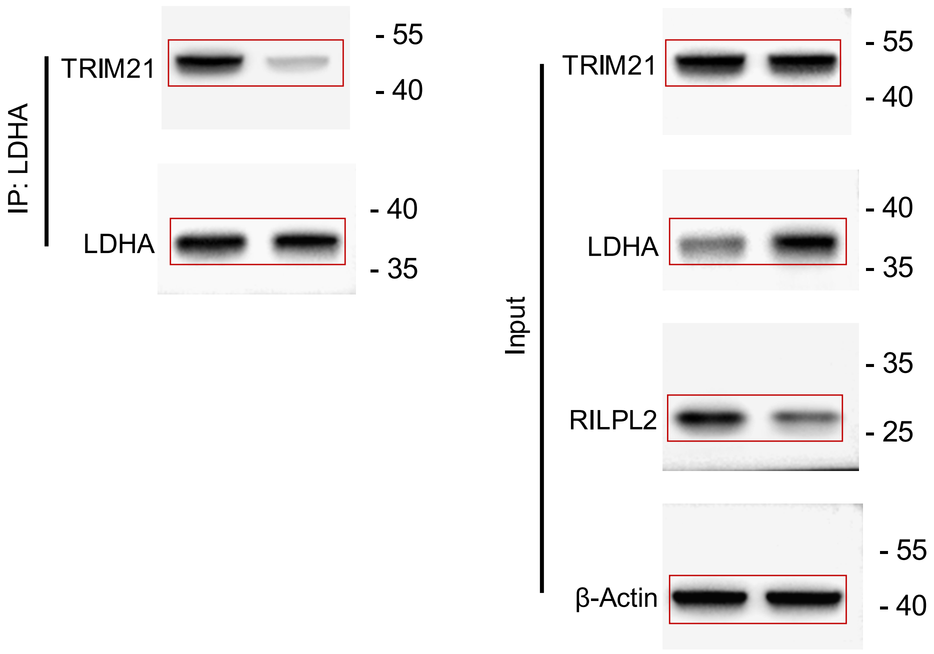

Fig. S9A

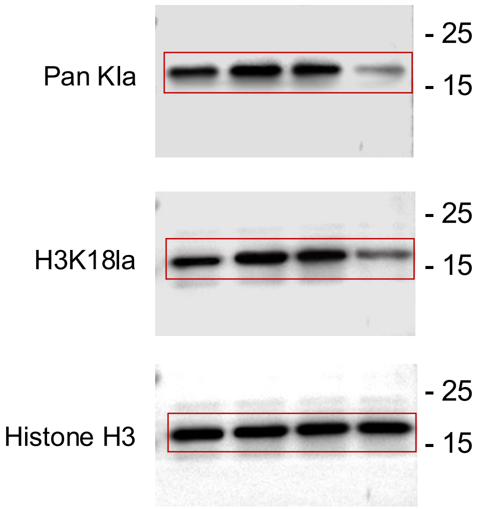

Fig. S9D

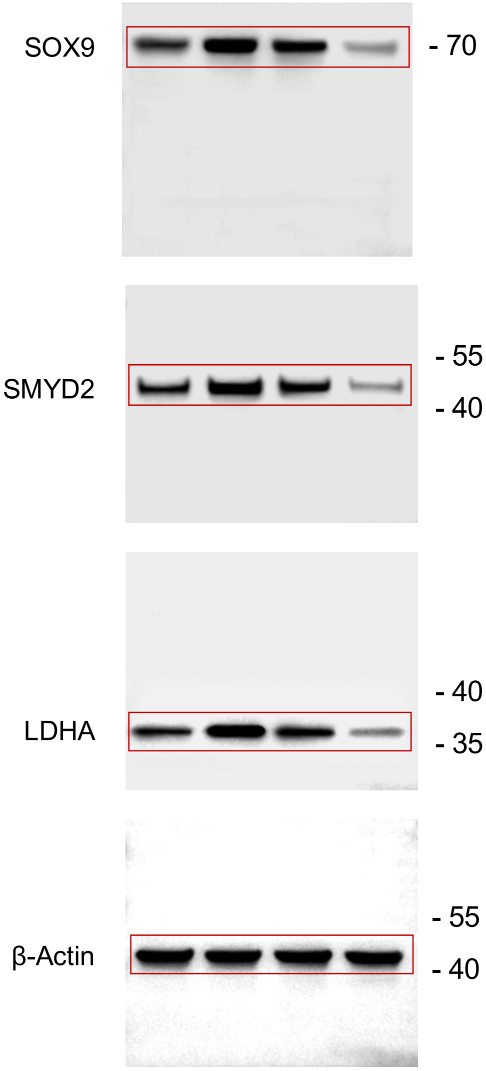

Supplement: Supplementary file 3 — full uncropped Gels and Blots image(s) [file 41419_2026_8808_MOESM3_ESM.pdf]
